# Combining specificity determining and conserved residues improves functional site prediction

**DOI:** 10.1186/1471-2105-10-174

**Published:** 2009-06-09

**Authors:** Olga V Kalinina, Mikhail S Gelfand, Robert B Russell

**Affiliations:** 1 EMBL Heidelberg, Meyerhofstrasse 1, 69117 Heidelberg, Germany; 2 Institute for Information Transmission Problems RAS, Bolshoi Karenty pereulok 19, Moscow, 127994, Russia

## Abstract

**Background:**

Predicting the location of functionally important sites from protein sequence and/or structure is a long-standing problem in computational biology. Most current approaches make use of sequence conservation, assuming that amino acid residues conserved within a protein family are most likely to be functionally important. Most often these approaches do not consider many residues that act to define specific sub-functions within a family, or they make no distinction between residues important for function and those more relevant for maintaining structure (e.g. in the hydrophobic core). Many protein families bind and/or act on a variety of ligands, meaning that conserved residues often only bind a common ligand sub-structure or perform general catalytic activities.

**Results:**

Here we present a novel method for functional site prediction based on identification of conserved positions, as well as those responsible for determining ligand specificity. We define Specificity-Determining Positions (SDPs), as those occupied by conserved residues within sub-groups of proteins in a family having a common specificity, but differ between groups, and are thus likely to account for specific recognition events. We benchmark the approach on enzyme families of known 3D structure with bound substrates, and find that in nearly all families residues predicted by SDPsite are in contact with the bound substrate, and that the addition of SDPs significantly improves functional site prediction accuracy. We apply SDPsite to various families of proteins containing known three-dimensional structures, but lacking clear functional annotations, and discusse several illustrative examples.

**Conclusion:**

The results suggest a better means to predict functional details for the thousands of protein structures determined prior to a clear understanding of molecular function.

## Background

Structural genomics, and the increased pace of structure determination by X-ray and NMR approaches makes methods to predict protein function from 3D structure of continuing importance. Proteins of known structure and unknown function are normally subjected to a battery of comparisons to find proteins adopting similar folds (DALI [1], SSAP [2], CE [3] and others) or containing recurring active-site residue constellations (SPASM [4], PINTS [5], Catalytic Site Atlas [6]). Proteins of similar structure can provide functional hints, since it is very often the case that proteins share structural and functional similarities in the absence of sequence similarity. Active site-only similarities (i.e. in the absence of overall fold similarity) are typically less revealing, but can sometimes suggest the presence of a convergently evolved catalytic machinery (e.g. the peptidase catalytic triad [7]) or binding sites for particular metals or ligands [8].

When comparative approaches fail to identify a clear similarity, or if such similarities are ambiguous – for instance, suggesting a possible weak functional similarity requiring confirmation – additional functional hints can come from analyses of the protein structure, similar structures, and what is typically a large collection of homologous sequences from the set of genomes now available. There are now several methods that exploit sequence conservation to identify putative functional sites in proteins, including ConSurf [9], approaches based on identification of 3D clusters of conserved residues [10], the evolutionary trace (ET) approach [11,12], correlated mutations [13], prediction of 3D motifs correlated with function [14], Jensen-Shannon entropy approach [15], algorithm based on contrasting global and local similarity matrices that interpret locality in terms of sequence [16] or structure [17]. The approaches differ in design, but share a unifying theme of using conserved amino acids, together with structural constraints such as location on the protein surface, as indicators of likely functional importance.

Some of the presented techniques make a special emphasis on using a protein structure for prediction. A number of methods identify interaction hot spots on different kinds of interaction interfaces [18-20]. Other methods concentrate on predicting pockets in protein structures, as they are possible ligand-binding sites [21-23], sometimes supplementing them with annotation derived from other available sources [24].

In contrast to those, several approaches, including our own [25], have attempted to exploit protein sequence alignments to determine those residues likely to confer specificity for a particular sub-function in a protein family [25-41]. Although they differ in algorithmic details, all approaches aim to use the statistics of a multiple sequence alignment to identify positions that correlate well with sub-families that account for a certain specific function. Sub-families are either explicitly given in advance (for instance taken from gene or protein annotation) or are predicted by the algorithm.

Here, we extend our previously derived approach for identifying specificity determining residues [25] to the problem of predicting protein functional sites (SDPsite). We combine routine predictions of conserved residues with those for specificity determinants, and use structural information to identify spatial clusters of the predicted important residues. SDPsite differs from structure-based methods in that the major part of the prediction is derived from the protein sequences. So, in theory, the method can also be applied in absence of structural information. The structure-based step filters out part of the predicted positions, thus leaving only the most reliable predictions, which can be useful in the design of experimental studies.

To test our method on a large scale, we developed two benchmark datasets of diverse enzyme families, using the Enzyme Classification (EC) system. Enzymes are the simplest class of proteins to benchmark as their functional annotations are well specified in databases. However, they are not necessarily representative of other protein functions that are less discretely characterized by precise catalytic machinery (e.g. protein recognition modules, etc.). In the absence of a reliable source of functional annotations for non-enzymes, we previously tested the presented approach on two examples, for which reasonable experimental data are available, the LacI family of the bacterial transcription factors and subtilisin-like proteases [42]. On these examples, SDPsite results have a sensitivity between 0.06 and 0.17, specificity between 0.43 and 0.75 and false positive rate between 0.007 and 0.05. Thus, in these anecdotal cases, SDPsite seems to miss a lot of truly functional amino acids, but still provides reliable predictions.

We also predicted the functional sites in 124 unannotated structures derived from Structural Genomics efforts. For the benchmark datasets, the success rate of our method (SDPsite) was 96–100%. We were then able to make confident functional site predictions for ~76% of a set of families lacking functional annotation.

## Results

### 1. Testing SDPsite on a benchmark set of enzymes with bound cognate ligands

We previously tested SDPsite on a number of protein families with known sites and compared the performance with several other approaches [42]. The results encouraged us to predict functionally important sites in poorly characterized protein families. Structural genomics projects now provide up to 20% of annual growth of the Protein data bank (PDB) and a greater coverage than ever before of the space of protein structures [43]. This has led to the current situation where hundreds of protein families include a protein with a known 3D structure, but no available functional information. These families are a perfect target for functional site prediction methods that use both sequence and structural information.

However, before applying our approach to families lacking functional information, we needed to benchmark the approach on a set of protein families that are well-characterised in terms of function. To do this we considered enzyme protein families from the Pfam database, for which there is a functional characterization scheme in the Enzyme Classification (EC) number system. This classification consists of four numbers denoting a hierarchical system that delineates enzyme function. We focused on families containing EC numbers differing in the last number, which normally accounts for the substrate specificity. Since protein families generally correspond to a single functional or structural domain, complications can arise for multi-domain proteins that correspond to a single EC number. We inspected the families manually to ensure that the catalytic operation for each EC number did indeed correspond to the domain considered. Thus each considered protein domain corresponds to a single EC number, thus catalyzing only one reaction (or one class of reactions), and presumably have only one active site.

To assess performance of SDPsite on these Pfam enzyme families, we generated two benchmark datasets. The first consisted of families containing proteins with at least two EC numbers differing in the last position; the second consisted of families containing only a single EC number. We refer to these datasets as *diverse *and *homogeneous *in the sections that follow. The rationale is that when one is predicting function and/or specificity, one does not known in advance whether or not there are multiple specificities in the family. These two datasets mimic both of these situations.

For all families we computed both specificity determining positions (SDPs) and conserved positions (CPs), then mapped them onto a 3D structure of one of the proteins of the family and extracted a portion of the two sets that forms a compact spatial cluster, as described previously [42] and in the Methods section. We designed several distance measures to assess the quality of the predictions. These were: 1) the minimal distance from the residues of each of the predicted sets (SDPs, CPs, best cluster) to a bound ligand; 2) the average distance to the ligand; 3) the diameter of the set; and 4) the average distance between residues of the set. We performed a Mann-Whitney test to assess the statistical significance of the best derived cluster, i.e. we tested if the set of the amino acid residues in the best cluster is significantly closer to the ligand than all residues in the protein. These data are given in Additional file 1.

We considered predictions to be successful if minimal distances were smaller than 5 Å and average distances smaller than 10 Å. We selected these thresholds based on inspection of known binding sites, and found that they capture characteristics of typical binding sites, which are normally 15–20 Å in diameter and typically some of the amino acids of the cluster contact the ligand directly. A small minimal distance and a large average distance means that the cluster is too sparse and does not define the active site well enough, but still a part of it is close to ligand and might be functional. Generally there is no correlation between either diameter or average distance within a predicted set of residues and the set's proximity to the active site.

As might be expected, predicted SDPs tend to be more sparsely distributed in the structure, compared to the more compact distribution of CPs. The best clusters are tightest, which is natural from their construction procedure, though the minimal distance suggests they are sometimes further away from the active site (even if the average distance is similar to CPs). We discuss these observations in more detail below.

#### a. The diverse dataset: protein families with at least two distinct EC numbers

**Figure 1:**
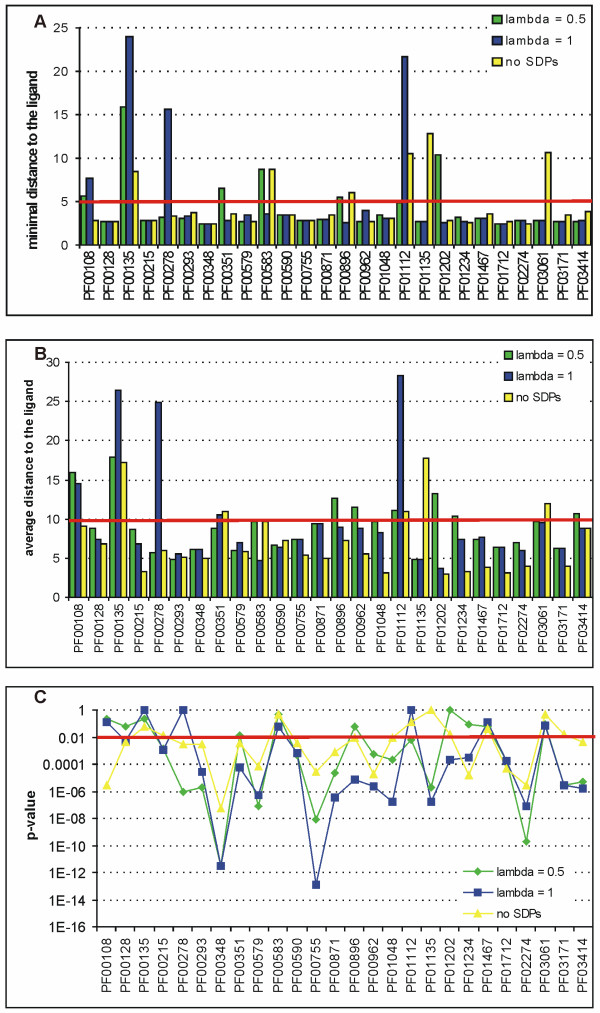
**Assessment of the prediction quality for the diverse dataset**. In each plot, the green and the blue bars represent SDPsite predictions with *λ *= 0.5 and *λ *= 1, respectively. Yellow bars represent prediction based solely on conserved positions. (a) Minimal distance from the best cluster to the bound ligand. (b) Average distance from residues of the best cluster to the bound ligand. (c) Significance of the average distance.

Application of all the filters described in the Methods section yielded 26 Pfam families (Table 1). SDPsite was applied in different ways, either ignoring SDPs thus mimicking the standard, conservation-based approaches, or including them when constructing the best cluster. For the inclusion of SDPs, we either gave them twice the weight as the CPs (*λ *= 0.5, *λ *being the relative weight of a CP to an SDP) or same weight (*λ *= 1). (Fig. 1). For details on the choice of the *λ *parameter, see Methods. In all but one of the considered families (Carboxylesterase, see below) at least one predictor performs well, and in the Asparaginase_2 family the average distance is slightly higher than 10 Å. This means that the best clusters are located in enzyme catalytic sites, and some of residues are in direct contact with the ligand. This result is significant (p < 0.01) for all but four, one of which is the Carboxylesterase family; for the other three the best cluster contains positions accounting for intersubunit contacts. The resulting implications for quaternary structure are discussed below.

**Table 1:** Statistics of the benchmark datasets: diverse dataset, two or more EC numbers per family

Family ID	Family name	# sequences	Alignment length	ECs	PDB	Bound ligand equivalent to natural substrate/product
PF00108	***Thiolase_N***	22	291	2.3.1.9 2.3.1.16 2.3.1.176	1NL7	Coenzyme A

PF00128	***Alpha-amylase***	54	673	2.4.1.4 2.4.1.7 3.2.1.10 3.2.1.20 3.2.1.70 3.2.1.98 3.2.1.93 3.2.1.141 5.4.99.16 5.4.99.15	2D3N	Glucose

PF00135	***COesterase***	129	889	3.1.1.1 3.1.1.3 3.1.1.7 3.1.1.8 3.1.1.13 3.1.1.59	1P0M	Choline ion

PF00215	***OMPdecase***	92	402	4.1.1.23 4.1.1.85	2CZE	Uridine-5'-monophosphate

PF00278	***Orn_DAP_Arg_deC***	55	220	4.1.1.17 4.1.1.18 4.1.1.19 4.1.1.20	1TWI	Lysine

PF00293	***NUDIX***	205	314	2.7.7.1 3.6.1.13 3.6.1.17 3.6.1.52 3.6.1.52 5.3.3.2	2DSC	Adenosine-5-diphosphoribose

PF00348	***Polyprenyl_synt***	16	289	2.5.1.10 2.5.1.29	2F8Z	Zoledronic acid, 3-methylbut-3-enyl trihydrogen diphosphate

PF00351	***Biopterin_H***	6	332	1.14.16.1 1.14.16.2 1.14.16.4	1MMK	5,6,7,8-tetrahydrobiopterin, beta(2-thienyl)alanine

PF00579	***tRNA-synt_1b***	41	402	6.1.1.1 6.1.1.2	1WQ4	Tyrosine

PF00583	***Acetyltransf_1***	244	150	2.3.1.1 2.3.1.4 2.3.1.48 2.3.1.57 2.3.1.59 2.3.1.82 2.3.1.87 2.3.1.88 2.3.1.128	1TIQ	Coenzyme A

PF00590	***TP_methylase***	22	247	2.1.1.98 2.1.1.107 2.1.1.130 2.1.1.131 2.1.1.132 2.1.1.133 2.1.1.152 2.1.1.151 4.2.1.75 4.99.1.4	1S4D	S-adenosyl-L-homocysteine

PF00755	***Carn_acyltransf***	22	867	2.3.1.6 2.3.1.7 2.3.1.21 2.3.1.137	1NDI	Coenzyme A

PF00871	***Acetate_kinase***	12	405	2.7.2.1 2.7.2.7 2.7.2.15	1TUY	Adenosine-5'-diphosphate

PF00896	***Mtap_PNP***	13	288	2.4.2.1 2.4.2.28	1V48	9-(5,5-difluoro-5-phosphonopentyl)guanine

PF00962	***A_deaminase***	17	475	3.5.4.4 3.5.4.6	1NDZ	1-((1r)-1-(hydroxymethyl)-3-(6-((3-(1-methyl- 1h-benzimidazol-2-yl)propanoyl)amino)-1h- indol-1-yl)propyl)-1h-imidazole-4-carboxamide

PF01048	***PNP_UDP_1***	16	276	2.4.2.1 2.4.2.3 2.4.2.28 3.2.2.4 3.2.2.9	1PK7	Adenosine

PF01112	***Asparaginase_2***	7	365	3.5.1.1 3.5.1.26	1SEO	Aspartic acid

PF01135	***PCMT***	9	232	2.1.1.77 2.1.1.36	1R18	S-adenosyl-L-homocysteine

PF01202	***SKI***	100	263	2.7.4.3 2.7.1.12 2.7.4.14 2.7.1.71 4.2.3.4	1WE2	Adenosine-5'-diphosphate

PF01234	***NNMT_PNMT_TEMT***	7	289	2.1.1.1 2.1.1.28 2.1.1.49	2AN4	S-adenosyl-L-homocysteine

PF01467	***CTP_transf_2***	66	302	2.7.7.1 2.7.7.3 2.7.7.14 2.7.7.15 2.7.7.18 2.7.7.39	1N1D	[Cytidine-5'-phosphate] glycerylphosphoric acid ester

PF01712	***dNK***	14	174	1.6.99.3 2.7.1.21 2.7.1.74 2.7.1.76 2.7.1.113 2.7.1.145	2A2Z	Uridine-5'-diphosphate, 2'-deoxycytidine

PF02274	***Amidinotransf***	32	455	2.1.4.1 3.5.3.6 3.5.3.18	2A9G	Arginine

PF03061	***4HBT***	153	102	3.1.2.2 3.1.2.23	1LO7	2-oxyglutaric acid, 2-aminoethanesulfonic acid

PF03171	***2OG-FeII_Oxy***	147	183	1.14.11.2 1.14.11.4 1.14.11.7 1.14.11.9 1.14.11.11 1.14.11.13 1.14.11.19 1.14.11.20 1.14.11.23 1.14.11.26 1.14.17.4 1.14.20.1 1.21.3.1	2FDJ	4-hydroxyphenacyl coenzyme A

PF03414	***Glyco_transf_6***	6	341	2.4.1.87 2.4.1.40	1LZJ	Succinic acid

Average values for all the above measures are given in Tables 2 and 3. Note that SDPs contribute to identification of the active site, although leading to prediction of a more disperse cluster. When no SDPs are predicted (last column), the average distance is smaller, because CPs form a more compact cluster in the active pocket. Unlike other methods that attempt to predict functional sites solely using the conservation of surface residues, SDPsite predicts a number of additional positions of potential importance. In 11 out of 26 families the best cluster is significantly (p-value < 0.01 in a Mann-Whitney test) closer to the ligand in both *λ *= 0.5 and *λ *= 1 scenarios, and in 20 out of 26 in at least one of them, whereas the CPs-only based prediction succeeded in only 15 families (Table 4). This indicates that the inclusion of SDPs in the prediction often leads to a significant improvement.

**Table 2:** Averages for the best cluster over all families from the Datasets, Å.

	Diverse dataset			Homogeneous dataset		
	*λ *= 0.5	*λ *= 1	No SDPs	*λ *= 0.5	*λ *= 1	No SDPs

minimal distance	4.22	5.37	4.56	3.81	2.95	4.84

average distance	8.89	9.95	7.14	9.83	8.39	8.47

**Table 3:** Sensitivity and false positive rate over all families from the Datasets, Å.

	Diverse dataset			Homogeneous dataset			Combined dataset		
	*λ *= 0.5	*λ *= 1	No SDPs	*λ *= 0.5	*λ *= 1	No SDPs	*λ *= 0.5	*λ *= 1	No SDPs

Sensitivity	0.13	0.14	0.06	0.13	0.16	0.07	0.14	0.15	0.07

False positive rate	0.03	0.02	0.008	0.05	0.04	0.01	0.04	0.03	0.01

**Table 4:** Assessment of SDPsite versions, diverse dataset.

Family	SDPsite (*λ *= 0.5 AND *λ *= 1)	SDPsite (*λ *= 0.5 OR *λ *= 1)	SDPsite (no SDPs)
PF00108	-	-	+
PF00128	-	-	+
PF00135	-	-	-
PF00215	+	+	-
PF00278	-	+	-
PF00293	+	+	+
PF00348	+	+	+
PF00351	-	+	+
PF00579	+	+	+
PF00583	-	-*	-
PF00590	+	+	-
PF00755	+	+	+
PF00871	+	+	+
PF00896	-	+	+
PF00962	-	+	+
PF01048	-	+	-
PF01112	-	+	-
PF01135	+	+	-
PF01202	-	+	+
PF01234	-	+	+
PF01467	-*	-	-*
PF01712	+	+	+
PF02274	+	+	+
PF03061	-*	-*	-
PF03171	+	+	-
PF03414	-	+	+

Accuracy	**0.42 (11/26)**	**0.78 (20/26)**	**0.58 (15/26)**

'+': successful prediction (average distance to ligand <10 Å, p-value in Mann-Whitney test against all amino acids of the protein < 0.01), '-*': moderately successful prediction (average distance to ligand <10 Å, p-value in Mann-Whitney test against all amino acids of the protein < 0.1), '-' otherwise.

For instance, in the Protein-L-isoaspartate(D-aspartate) O-methyltransferase (PCMT, PF01135) and Thioesterase (4HBT, PF03061) families, the CP-based cluster is located distant from the active site, whereas addition of SDPs rescues the prediction leading to the correct site. In the C-terminal domain of Pyridoxal-dependent decarboxylases (Orn_DAP_Arg_deC, PF00278), bacterial transferase hexapeptide (Hexapep, PF00132) and Asparaginase (Asparaginase_2, PF01112) families, SDPs rescue the cluster for *λ *= 0.5. In contrast, for the Shikimate kinase family (SKI, PF01202) a heavier reliance on SDPs skews the prediction, whereas more equal considerations of SDPs and CPs, or of CPs only, perform considerably better.

The Carboxylesterase (COesterase; PF00135) family is the only clear failure of the method, i.e. its active site is not identified by either variant of the method. Even the catalytic triad, Ser198, His438 and Glu197 (numbering from ChlE_Human), is not among either SDPs or CPs. The fact that the catalytic residues are not conserved in the alignment is puzzling. This could be because the alignment from Pfam contains many uncharacterized paralogs from C. elegans and D. melanogaster, which could perform a different function or be non-functional. Indeed, catalytic residues are often substituted in these proteins: Ser198 to alanine, asparagine, glycine or valine, His438 to asparagine, glutamic acid, leucine, lysine, tyrosine or valine, and Glu197 to asparagine, glutamic acid, glutamine, histidine, isoleucine, proline, threonine, tryptophane or tyrosine. Such changes mean that these residues are ignored in the prediction procedure, and highlights the need for some caution when building alignments to predict function.

We overlook details of quaternary structure when making predictions, and this can have interesting consequences, as discussed previously (e.g. ref. [25]). For instance, for the Thiolase N-terminal domain (Thiolase_N, PF00108) family, we found the minimal and the average distance to be rather large. From the structure of a protein from this family (biosynthetic thiolase from *Z. ramigera*, 1NL7), it is evident that the best cluster is located on the contact interface between two subunits of a dimer. Indeed, the minimal and the average distance to the second subunit are 2.73 Å and 6.90 Å, respectively. The second best cluster is, however, in the active pocket with the distances below the threshold. The family of Gcn5-related acetyltransferases (Acetyltransf_1, PF00583) is a similar case: for *λ *= 0.5 the best cluster is located on subunit contact interface and the second best in the active site, for *λ *= 1 vice versa. This highlights the need to consider quaternary structure explicitly when making and interpreting predictions using this or similar approaches.

A natural question is how well the predicted grouping of the sequences agrees with the EC numbers of the proteins considered. For most families there was a good agreement, with EC numbers segregating into discrete branches of the trees derived from the alignments. There were two families where proteins with one EC number would split between two groups that contain proteins with other EC numbers (alpha-amylase, PF00128, and polyprenyl synthetase families, PF00348). For both, the same enzymatic activity seems to evolve independently on two separate branches of the phylogenetic tree.

#### b. The homogeneous dataset: protein families with a single EC number

**Figure 2:**
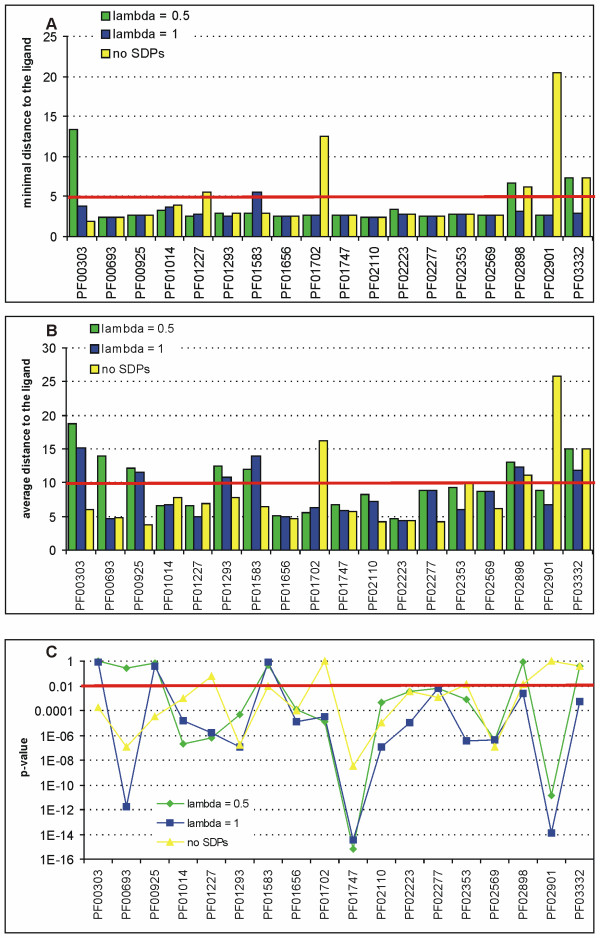
**Assessment of the prediction quality for the homogeneous dataset**. Color code as in Fig. 1. (a) Minimal distance from the best cluster to the bound ligand. (b) Average distance from residues of the best cluster to the bound ligand. (c) Significance of the average distance.

The 18 families with a single EC number are listed in Table 5. Again, we applied SDPsite with *λ *= 0.5, *λ *= 1 and without prediction of SDPs (Fig. 2). For all studies families, except Eukaryotic phosphomannomutases (PMM, PF03332), at least one of these variants puts the best cluster to the active site of the enzyme according to the described criteria. For 9 out of 18, the best cluster identified by either procedure is located in the active site. These results are significant (p < 0.01) for all families, except Adenylylsulphate kinases (discussed below).

**Table 5:** Statistics of the benchmark datasets: homogeneous dataset, strictly one EC number per family

Family ID	Family name	# sequences	Alignment length	EC	PDB	Bound ligand equivalent to natural substrate/product
PF00303	Thymidylat_ synt	19	384	2.1.1.45	2G8O	2'-deoxyuridine 5'-monophosphate, 10-propargyl-5,8-dideazafolic acid

PF00693	Herpes_TK	15	305	2.7.1.21	1VTK	Adenosine-5'-diphosphate, thymidine-5'-phosphate

PF00925	GTP_ cyclohydro2	16	193	3.5.4.25	2BZ0	Phosphomethylphosphonic acid guanylate ester

PF01014	Uricase	17	196	1.7.3.3	2FXL	1-(2,5-dioxo-2,5-dihydro-1h-imidazol-4-yl)urea

PF01227	GTP_ cyclohydroI	16	107	3.5.4.16	1A8R	Guanosine-5'-triphosphate

PF01293	PEPCK_ATP	12	495	4.1.1.49	1YTM	Adenosine-5'-triphosphate, oxalic acid

PF01583	APS_kinase	20	166	2.7.1.25	1M7G	Adenosine-5'-phosphosulfate, adenosine-5'-diphosphate-2',3'-vanadate

PF01656	CbiA	80	372	6.3.3.3	1A82	Adenosine-5'-triphosphate, 7,8-diamino-nonanoic acid

PF01702	TGT	13	256	2.4.2.29	1Q2S	9-deazaguanine

PF01747	ATP- sulfurylase	19	397	2.7.7.4	1G8H	Adenosine-5'-phosphosulfate, pyrophosphate 2-

PF02110	HK	9	282	2.7.1.50	1ESQ	Adenosine-5'-triphosphate, 4-methyl-5-hydroxyethylthiazole phosphate

PF02223	Thymidylate_ kin	26	209	2.7.4.9	1E9E	Adenosine-5'-diphosphate, thymidine-5'-phosphate

PF02277	DBI_PRT	28	398	2.4.2.21	1L5L	7-alpha-d-ribofuranosyl-purine-5'-phosphate, nicotinic acid

PF02353	CMAS	12	304	2.1.1.79	1KPI	S-adenosyl-Ll-homocysteine

PF02569	Pantoate_ ligase	7	311	6.3.2.1	2A86	Adenosine monophosphate, beta-alanine

PF02898	NO_synthase	8	374	1.14.13.39	1Q2O	L-n(omega)-nitroarginine-2,4-L-diaminobutyric amide

PF02901	PFL	11	734	2.3.1.54	1MZO	Pyruvic acid

PF03332	PMM	9	248	5.4.2.8	2FUE	Alpha-d-mannose 1-phosphate

The remaining nine families, for which active sites were not identified by all variants of SDPsite, can be split into four categories: (1) *λ *= 0.5 fails (Thymidylate synthases, Thymidylat_synt, PF00303; thymidine kinases from Herpesviridae, Herpes_TK, PF00693); (2) both *λ *= 0.5 and *λ *= 1 fail (GTP cyclohydrolases II, GTP_cyclohydro2, PF00925; Phosphoenolpyruvate carboxykinases, PEPCK_ATP, PF01293; Adenylylsulphate kinases, APS_kinase, PF01583); (3) CP-only prediction fails (Queuine tRNA-ribosyltransferases, TGT, PF01702; Pyruvate formate lyases, PFL, PF02901); and (4) at least two of the three fail (oxygenase domain of Nitric oxide synthases, NO_synthase, PF02898; Eukaryotic phosphomannomutases, PMM, PF03332).

SDPs are expected to perform worse for this dataset, because in theory there are no specificity differences within each family as defined by the EC system. However, CP-based clusters significantly skew the predictions in two cases out of eighteen. One explanation of this observation could be inaccuracy or ambiguity in the assignment of the EC numbers, which would mean that we have identified some real differences the specificity. It might also be that a substitution to an amino acid with similar properties (e.g. size, polarity, charge) occurred, which can perform an equivalent enzymatic role. Another possibility is the functional convergence to a single specificity at the molecular level: SDPs indicate different residues that distant species or distant paralogs evolved to perform the same function (e.g. [44]). Alternatively, as discussed below, SDPs may indicate differences specific to certain phylogenetic clades.

Similarly to the diverse dataset, there are two families for which the best cluster is located on the subunit contact interface: Thymidylate synthases and Adenylylsulphate kinases. The minimal and average distances to the second subunit of the dimer are 3.30 Å and 5.73 Å for Thymidylate synthases and 2.65 Å and 6.55 Å for Adenylylsulphate kinases. For both, the second best cluster is in the active site.

For two families, all three versions of SDPsite produce poor results. For the oxygenase domain of Nitric oxide synthases the average distance of the residues from the best cluster to the ligand is between 10 and 15 Å for all three versions, and for Eukaryotic phosphomannomutases it even exceed 15 Å in two out of three versions. However, for both families, the second best cluster is located in the active site. In the case of the oxygenase domain of Nitric oxide synthases the alignment contains only 8 sequences, which split into phylogenetic groups of insects and vertebrates. These groups are too distant, and it seems that the method cannot remove the phylogenetic trace completely, thus producing many SDPs that are probably not functionally important. A more representative set of sequences may improve the predictions and probably place the best cluster in the right position. For the Eukaryotic phosphomannomutases, the best cluster is located in the core domain close to the magnesium ion, which is a part of the active site, but distant from the substrate in the open conformation of the protein.

The means of the minimal and the average distances to the ligand and the significance of the average distance are summarized in Table 2. It is not surprising that giving SDPs and CPs equal weight (*λ *= 1) leads to better results than *λ *= 0.5 for this dataset: since all the proteins have the same EC number, one might not expect to find anything accounting for differences in specificity among these proteins. As discussed above, the identified SDPs could, instead, reflect different ways that different groups of proteins evolved to perform the same function. Again, building the best cluster from only CPs does not improve the prediction quality, and all the distances used as the performance measures are larger than for *λ *= 1. This is another indication that proteins, even though they have the same function and specificity, have evolved quite different ways to perform it, and this must be taken into account when predicting functional sites.

In contrast to the diverse dataset, the success rate is similar for different versions of SDPsite (Table 6). In 10 out of 18 families the best cluster identified by taking into account both CPs and SDPs is significantly closer to the ligand (p-value < 0.01 in Mann-Whitney test), which indicates that specificity determinants predicted for homogeneous families, even when they do not illuminate the binding of a specific ligand, play some other important role in their function. In this regard, it is important to stress that this dataset is only homogenous as defined by EC numbers, and it is well established that these do not always uniquely define molecular function [45]. In other words, substantial sequence and structure diversity is possible even among sets of protein sharing the same EC number.

**Table 6:** Assessment of SDPsite versions, homogeneous dataset.

Family	SDPsite (*λ *= 0.5 AND *λ *= 1)	SDPsite (*λ *= 0.5 OR *λ *= 1)	SDPsite (no SDPs)
PF00303	-	-	+
PF00693	-	+	-
PF00925	-	-	+
PF01014	+	+	+
PF01227	+	+	-
PF01293	-	-	+
PF01583	-	-	+
PF01656	+	+	+
PF01702	+	+	-
PF01747	+	+	+
PF02110	+	+	+
PF02223	+	+	+
PF02277	+	+	+
PF02353	+	+	-
PF02569	+	+	+
PF02898	-	+	-
PF02901	+	+	-
PF03332	-	+	-

Accuracy	**0.61 (11/18)**	**0.78 (14/18)**	**0.55 (10/18)**

'+': successful prediction (average distance to ligand <10 Å, p-value in Mann-Whitney test against all amino acids of the protein < 0.01), '-' otherwise.

For example, the alignment of the queuine tRNA-ribosyltransferase family contains 9 bacterial and 4 archaeal proteins. When predicting SDPs, SDPsite clearly divides the family into these two groups. A closer analysis of the predicted SDPs reveals that some positions that are conserved within the two groups but differ between them bind substrate or tRNA in the bacterial enzyme from Zymomonas mobilis [46]. For example Cys 158 binds queuine precursor (substituted to proline in archaea), and Arg 286 binds tRNA (substituted to leucine).

#### c. Overall performance in the benchmark and guidelines for predictions

We analyzed the performance of SDPsite by calculating, for the diverse, homogenous and combined datasets, sensitivity (the ratio of number of true positives to the number of true positives plus false negatives) and false positive rate (ratio of number of false positives to the number of false positives plus true negatives) (Table 3). For a perfect prediction, sensitivity should be close to 1 and the false positive rate close to 0. As a gold standard set of residues in active sites, we considered all amino acids located within a distance of 10 Å from the bound ligand. Note that not all these residues are functionally important, which makes the reported false positive rate lower than it would be if we had perfect information on real functional importance of all residues in the proteins considered.

In all datasets the inclusion of SDPs in the predictions leads to a higher sensitivity. There is no significant difference between the diverse and homogeneous datasets, though this is probably due to the diverse nature of the underlying EC and sequence data as mentioned above: functional diversity is also likely present in the homogeneous dataset making SDPs beneficial to the prediction of functional sites.

As discussed above for the phosphomannomutase family, proteins often undergo conformational changes upon binding ligands, which means that any method tested on structures in complex with a ligand might unfairly profit from the use of a bound structure, which will differ from the unbound or *apo *form of the protein. To test for this effect, we identified 16 apo protein structures (of 26) from the diverse dataset and 14 (of 18) from the homogeneous dataset and found no significant difference in the predicted residues. This effectively means that the method is relatively insensitive to minor conformational changes that occur upon binding. It is worth noting, however, that the enzyme sites considered here might not be representative of other types of interactions that can lead to more substantial conformational re-arrangements (e.g. protein-protein interactions), or contain much larger functional sites than the tight constellations of functional residues normally found in enzymes.

The results from the above benchmark provides a guide for how to interpret predictions, which we used for the unannotated families below. Based on inspection of the results, we found the ratio of the total number of predicted SDPs and CPs to the length (i.e. (SDP+CP)/length) of alignment to be a useful measure of performance. For 29 of 44 families this ratio was below 0.2. For 17 of these 29 families all three versions of SDPsite predict the best cluster with an average distance to ligand less than 10 Å, and for 8 of these the p-value is < 0.01 in all three versions of the method. However, the set of families with successful predictions is not enriched with those with low (SDP+CP)/length ratio compared to all predictions. We also calculated the ratio of the number of residues in the best cluster to the length (best cluster/length) of alignment and applied the cutoff of 0.1. For 35 of total 44 (80%) families the best cluster/length ratio is below 0.1, whereas among families, for which average distance is <10 Å, and p-value < 0.01 for all three versions of SDPsite, this fraction is 10 out of 12 (83%). Thus it is practical first to consider predictions with a low best cluster/length. Still, as we discuss below, even more disperse predictions can lead to interesting insights.

As noted above, sometimes the best cluster can be situated on the inter-subunit contact interface, and the second best cluster in the active site. No clear strategy can be suggested to distinguish these situations in the absence of prior knowledge. In practice one should analyze the composition of the best cluster for the presence of amino acids typical for enzyme active sites (potential electron donors/acceptors) or for protein-protein contact interface (hydrophobic patches, etc.). These considerations, however, tend to be family-specific and thus cannot be included into a prediction algorithm intended for a general use.

It is worth noting that SDPsite performs best when the alignment is sufficiently long, the sequences are sufficiently diverse in terms of sequence identity and the phylogenetic tree is biologically sound. As a guideline, it performs best with alignments of at least 50 positions, and at least 10 proteins with identities between 30 and 90%. The diversity of possible sequences sets and functions for which SDPsite is applicable is great. From our benchmark, we were unable to distinguish between a single-EC and a multiple-EC family in a blind test. This is probably the major drawback of the method: in absence of additional information, one cannot conclude whether the identified SDPs pinpoint the real functional diversity within the family, or simply reflect the phylogenetic trace. It is impossible to say which of the three functional site prediction approaches is generally best. In practice, one should always run all three approaches, and then interpret based on what, if anything, is known about the function, and how the functional sites look on the structure.

### 2. Application to protein families lacking functional annotation

We focused on 193 Pfam families that included at least one protein with resolved 3D structure, and where all structures come from Structural Genomics Projects. After removing families with fewer than 6 sequences in the Pfam seed alignment, we were left with 124 (Additional file 2), for which potential functionally important sites could be identified. Of these, 54 families include potential or proven enzymes; 5 are transcription factors; 3 are involved in translation; 15 participate in various cellular processes in a fashion that is not completely understood; and the functional role for 47 is unknown. The full description of the predictions is available from the SDPsite web-site. We calculated SDP+CP/length and best cluster/length ratios for these families. For 50 of 124 families SDP+CP/length ratio was below 0.2 and the predicted cluster lies on the surface or in a pocket of the protein structure. For another 44 families predictions were weaker, but acceptable: SDP+CP/length ratio exceeds 0.2, but the cluster still lies in a pocket or on the surface. For the remaining 30, no reasonable predictions were made. Taken together, 76% of the predictions seem to provide a reasonable hint about the location of the actual functional site of the protein. The best cluster/length ratio is below 0.1 in 91 of 124 (73%) families. This fraction is lower than for the benchmark dataset, which can be due to the fact that the considered dataset includes proteins of various functions as opposed to the benchmark that consists solely of enzymes. The predictions made for these 91 families are the most promising candidates for experimental validation. Five promising examples are discussed below. These examples were chosen according to the criteria above, and our own visual inspection of the results.

#### a. YCII-related domain (PF03795)

This family was first identified during an analysis of the *Streptomyces coelicolor *genome by a domain hunting process [47]. The authors confined the annotation to a remark that it is "probably enzymatic". The study of this domain continued, when the first (and to date only) structure of a protein from this family, HI0828 from *Haemophilus influenzae*, was solved (PDB ID 1mwq, [48]). The protein was shown to adopt a ferrodoxin-like *α*/*β*-fold, and a catalytic mechanism involving a histidine-aspartate pair was proposed. However, biochemical assays have to-date failed to suggest a substrate for the enzyme.

**Figure 3:**
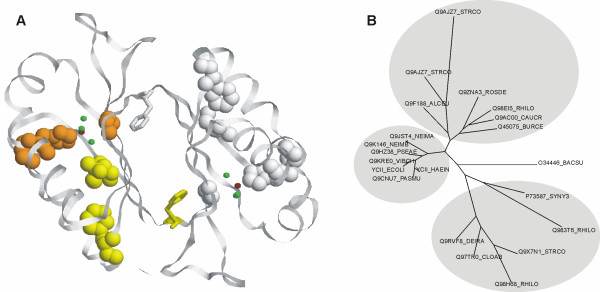
**A. Structure of HI0828 from *Haemophilus influenzae *(1 mwq)**. SDPs are marked yellow, CPs are marked orange, best cluster is shown in spheres. Cl ions are shown in green, Zn ions in brown. **B. Phylogenetic tree of the YCII-related domain (PF03795) family**. The predicted specificity groups are shown as gray ovals.

SDPsite identifies a small cluster of potentially important residues (Fig. 3a), located in a pocket, which contains a coordinated ZnCl_3_, a ligand supposed to play a role in the catalysis. The method splits the family into three specificity groups (Fig. 3b). As the family includes a number of paralogues from the same species, which fall into different specificity groups, the identified sub-groups might reflect the real specificity differences within the family. The predicted SDPs might thus account for binding of different substrates in the active pocket.

#### b. CobW/HypB/UreG, nucleotide-binding domain (PF02492), and Cobalamin synthesis protein cobW C-terminal domain (PF07683)

These two protein families represent the two domains of CobW protein, a hypothetical protein, involved in cobalamin biosynthesis and possibly required to store cobalt ions as an intermediary between the cobalt transport and chelation systems [49]. The N-terminal part of CobW is a member of PF02492, which also contains nucleotide-binding domains of HypB and UreG, GTPases involved in binding of nickel to apoenzymes [50,51]. The only known 3D structure in both families belongs to a hypothetical protein YjiA from *E.coli *[31] (PDB ID 1nij). YijA is a homolog of CobW and has the same domain structure, but a much shorter linker between the domains, and also lacks the histidines required for metal binding, and thus cannot serve as a metal repository. YijA is believed to be a GTP-dependent regulator, however its biological role is unclear [52]. The N-terminal domain of YjiA (which corresponds to PF02492) has a fold typical to P-loop NTP-binding proteins, and a number of motifs responsible for GTP binding can be found in it. The C-terminal domain has a ferrodoxin-like fold.

**Figure 4:**
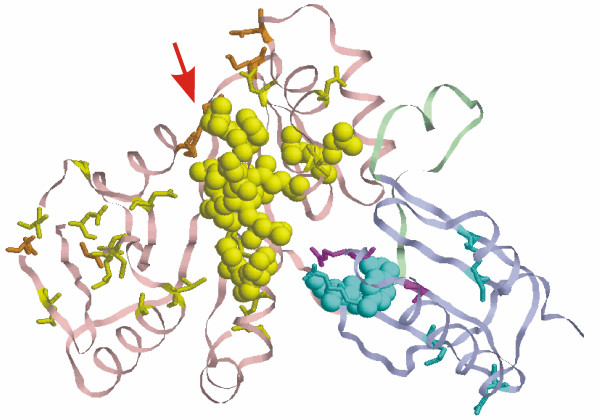
**Structure of YjiA from *E.coli ***(1nij). N-terminal domain is shown in pink, linker is shown in light green, C-terminal domain is shown in light blue. SDPs are marked yellow, CPs are marked orange in the N-terminal domain and cyan and magenta, respectively, in the C-terminal domain, best cluster is shown in spheres. The red arrow indicates the position of the nucleotide-binding pocket.

SDPsite identified two potential functionally important sites, one for each domain (Fig. 4). Both clusters are exposed to solvent and the cluster in the N-terminal domain is located close to the possible nucleotide-binding pocket, identified by Walker A and B motifs, as discussed in ref. [49] (arrow in Fig. 4). The arrangement of the clusters suggests their possible role in communication between the two domains. Indeed, in the structure used the minimal distance between residues of the N-terminal and the C-terminal domain cluster is 5.5 Å. As the domains are rather mobile relative to each other due to the flexible linker, under certain conditions the two clusters could potentially interact directly.

#### c. PHP domain (PF02811)

This family corresponds to a catalytic domain with a phosphoesterase activity found both as a stand-alone protein and fused to DNA polymerase domains. PHP domains are often found in the N-terminal part of bacterial DNA polymerase III *α *subunit and in the C-terminal part of DNA polymerase of the X family in some archaea. In this role, the PHP domain is proposed to hydrolyze pyrophosphate, shifting reaction equilibrium to polymerization [53]. The family also includes a number of tyrosine-protein phosphotases and histidinol phosphatases and many uncharacterized proteins. 3D structures are available for two proteins of the family, both with unknown function: YcdX from *E.coli *and tm0559 from *Thermotoga maritima*.

**Figure 5:**
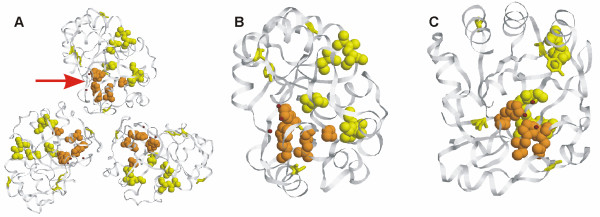
**Structure of YcdX from *E.coli ***(1m68). **A**. YcdX trimer. Putative location of the active site is indicated with an arrow. SDPs are marked in yellow, CPs in orange, the best cluster is shown in spheres. **B**. YcdX monomer. Side view. **C**. YcdX monomer. Front view.

We mapped the predicted positions on the structure of YcdX from *E.coli *[54] (PDB code 1m68) (Fig. 5), since the predicted clusters in all available structures are formed by equivalent residues (data not shown). YcdX is usually present as a trimer in solution, each monomer possessing its own catalytic site [54] (Fig. 5A, B and 5C show a closer view of one monomer of the complex). A cluster of three zinc ions is located in a cleft of the structure, indicating a possible location of the active site (marked with an arrow in Fig. 5A). The predicted cluster of functionally important residues lies close to the zinc cluster and has a layered form: a more compact layer of CPs and a fuzzier layer of SDPs. CPs might represent the catalytic core of the active site and SDPs, a less spatially defined recognition periphery. Some SDPs even protrude to the back of the monomer, where they can participate in the ligand recognition in another active site.

**Figure 6:**
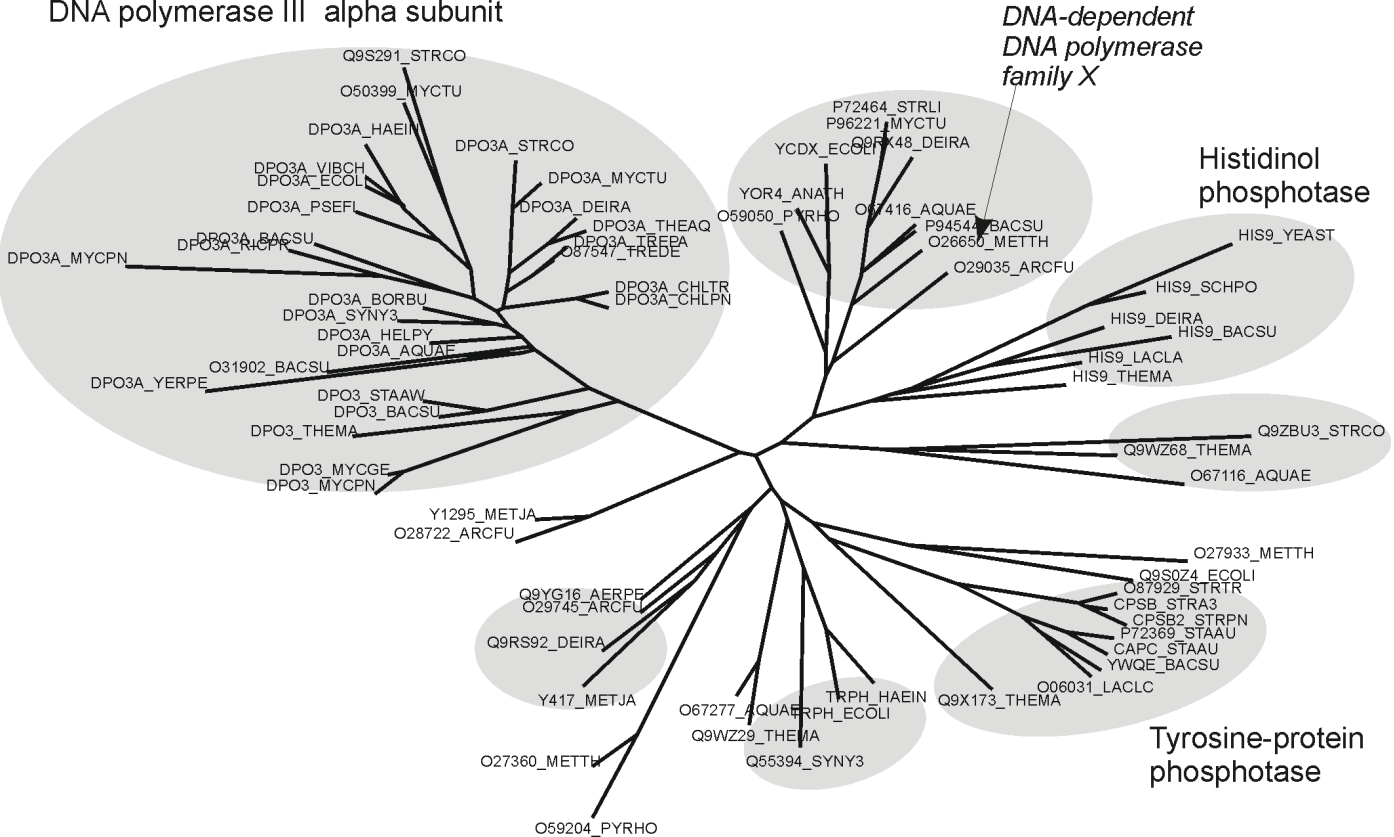
**Phylogenetic tree of the PHP domain (PF02811) family**. Specificity groups are shown as gray ovals. The predominant annotation for certain groups is indicated beside them. When only one protein of a group has a functional annotation, it is put in italics and indicated by an arrow.

It was previously noted [53] that proteins of the PHP family appear as single domain proteins or as domains in multi-domain proteins involved in a variety of cellular processes, possibly exhibiting diverse specificity. Indeed, one can find proteins annotated as "DNA polymerase III subunit alpha", "DNA-dependent DNA polymerase family X", "Histidinol-phosphatase", "Tyrosine-protein phosphatase" and "Protein trpH" in this family. Many of the family members are still uncharacterized. Along with the identification of a cluster of possible functionally important residues, SDPsite extracts a number of specificity groups, which are shown on the phylogenetic tree on the family in Fig. 6. Proteins with similar annotation always fall into same group, and, with a certain degree of caution, this annotation can be transferred onto other proteins of the same group. For example, YcdX falls into the same group with a sequence from *Methanobacterium thermoautotrophicum *(O26650), which is annotated as a DNA-dependent DNA polymerase family X, known to be involved in DNA repair. Analysis of gene expression level in stress conditions indicates that YcdX might also be involved in DNA repair [55].

#### d. Possible lysine decarboxilase (PF03641)

**Figure 7:**
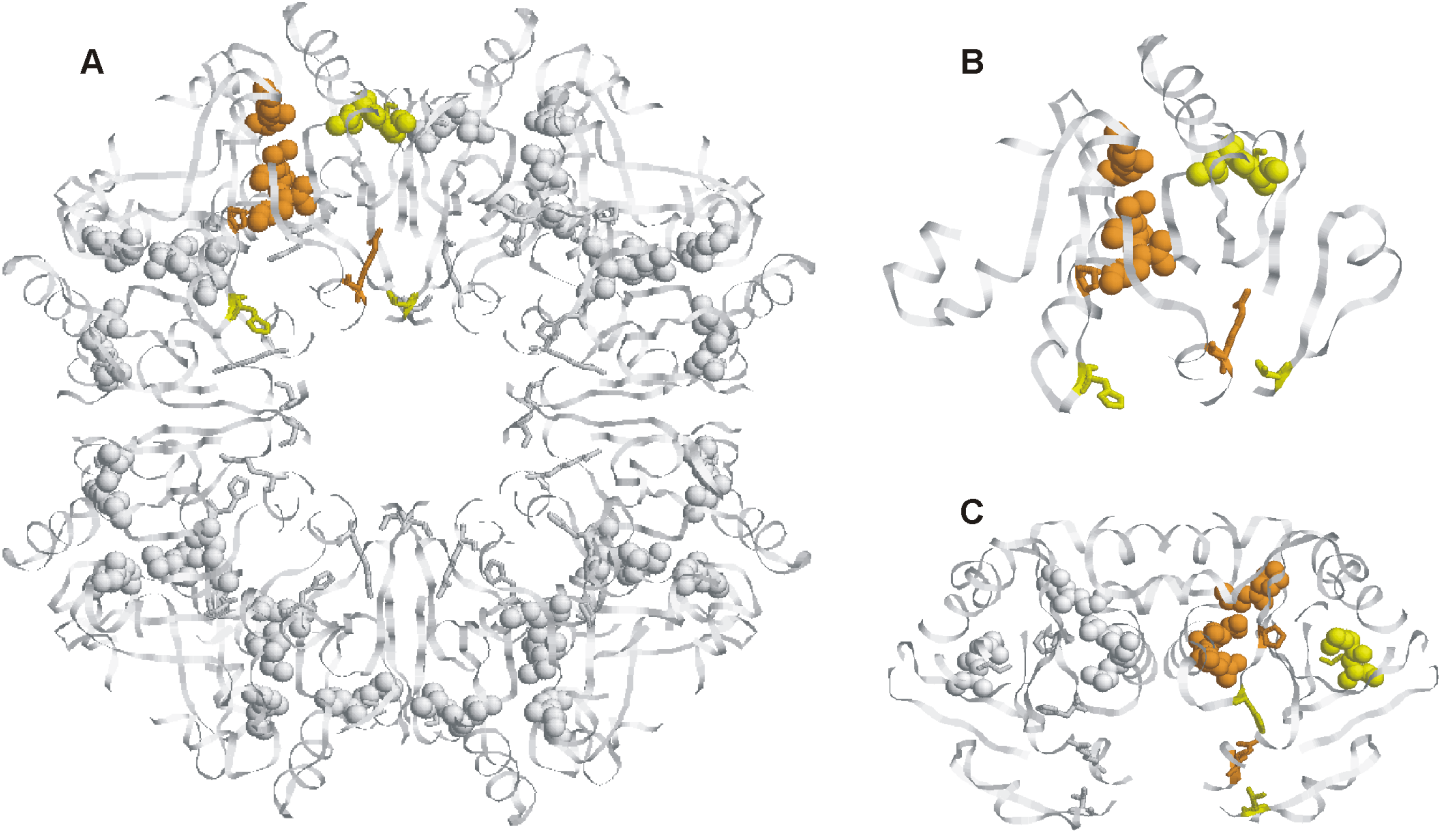
**Structure of YvdD from *Bacillus subtilis ***(1t35). **A**. YvdD octamer. Suggested biological unit (from PDB entry 1t35). The color coding is as for Fig. 5. **B**. YvdD monomer. **C**. YvdD dimer. Note that the active site might be located between the two subunits.

According to Pfam, this family includes proteins annotated as lysine decarboxylases, but the evidence for this annotation is not clear. Three structures from this family are available (YvdD from *Bacillus subtilis*, 1t35; tm1055 from *Thermotoga maritima*, 1rcu; and at5g11950 from *Arabidopsis thaliana*, 1ydh), none of them with clear functional annotation. One can clearly see a cleft lined with CPs with SDPs on its periphery (Fig. 7A, B and 7C show different possible oligomeric states), which could form an active pocket. It is also possible that there is one active pocket per every two subunits (see Fig. 7C).

#### e. YqeY-like proteins (PF09424)

**Figure 8:**
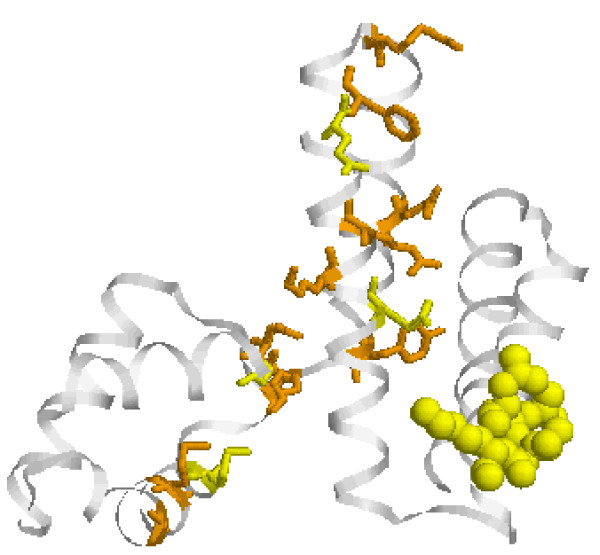
**Structure of YqeY from *Bacillus subtilis ***(1ng6). The color coding is as for Fig. 5.

YqeY-like proteins are widespread, but the largest portion of the family comes from bacteria (341 sequences vs. 17 in Eukarya, 1 in viruses and none in Archaea). Predominantly, there is only one copy per genome. Despite the great number of proteins in the family, their function remains unclear. A number of SDPs and CPs were identified in this protein (Fig. 8). The best cluster is a tight cluster of four SDPs at the C-terminal end of the second *α*-helix, and exposed to solvent. This positioning might suggest its involvement in some protein-protein interactions. The functional role of these residues, if any, remains to be tested.

### 3. Comparison to other functional site prediction methods

We compared the performance of SDPsite to several other techniques which also aim to predict functionally important residues from protein sequence and structure information: 1) the Evolutionary Trace (ET) method [11,12], 2) the ConSurf method [9], and 3) the three methods from the TreeDet package [30]. To produce predictions with the ET method, we used the TraceSuite II server . For ConSurf we used the Rate4site implementation [56], which assigns an evolutionary rate to each position of the alignment. The more a position is conserved, the more important for the function it is considered to be. As the algorithm produces a rating, rather than a set of functionally important positions, we considered the two most conserved of the nine output categories as the prediction.

We used the two benchmark datasets and the assessment procedure described in the above to assess the predictions of different methods. The minimal distance to the ligand, the average distance and the significance of the average distance are presented in Additional file 3. The predictions of the ET method usually show low minimal distance to the ligand, but due to the fact that ET renders too many positions as potentially important (up to 60% of the alignment length), which is impractical in experimental studies. We performed Mann-Whitney tests comparing the distribution of distances for the predictions produced by SDPsite to various other methods (Table 7). In 32 and 34 of 44 cases at least one of the three versions of the method (*λ *= 0.5, *λ *= 1 or no SDPs) performed significantly better (p-value < 0.01) than ET. and Rate4Site, respectively. FASS, MB and S methods perform well, but frequently identify too few amino acids to make the test reliable, or are not applicable at all due to the methods' limitations. SDPsite thus achieves a good balance between the quality and quantity of the predicted residues and the general applicability of the method to further investigations.

**Table 7:** Assessment of different methods.

Family	ET	Rate4site	FASS	MB	S
PF00108	C	C	-	-	no MW
PF00128	-	++C	n/a	n/a	n/a
PF00135	-	-	n/a	n/a	n/a
PF00215	-	++	no MW	-	no MW
PF00278	+	+C	-	-	no MW
PF00293	++C	+	n/a	n/a	n/a
PF00303	C	C	no MW	+	0
PF00348	++C	++C	-	++C	-
PF00351	+	+	n/a	n/a	n/a
PF00579	++	++	-	+C	no MW
PF00583	-	-	n/a	n/a	n/a
PF00590	++	++	no MW	-	-
PF00693	+C	+C	n/a	n/a	n/a
PF00755	++C	++C	n/a	n/a	n/a
PF00871	++C	++C	n/a	n/a	n/a
PF00896	+	-	n/a	n/a	n/a
PF00925	C	C	0	-	0
PF00962	++C	+C	no MW	++C	C
PF01014	++C	++C	no MW	++C	0
PF01048	+C	+C	-	-	-
PF01112	-	+	n/a	n/a	n/a
PF01135	++	++	n/a	n/a	n/a
PF01202	-	-	no MW	+	0
PF01227	+	-	-	-	0
PF01234	+C	+C	n/a	n/a	n/a
PF01293	+C	C	n/a	n/a	n/a
PF01467	-	-	0	-	0
PF01583	-	-	no MW	-	0
PF01656	++C	++C	no MW	-	no MW
PF01702	++	++	n/a	n/a	n/a
PF01712	++C	C	n/a	n/a	n/a
PF01747	++C	++C	++C	++C	-
PF02110	++C	+C	n/a	n/a	n/a
PF02223	++C	+	no MW	-	0
PF02274	++C	++C	-	C	-
PF02277	C	C	no MW	-	0
PF02353	++	++	n/a	n/a	n/a
PF02569	++C	C	n/a	n/a	n/a
PF02898	-	+	n/a	n/a	n/a
PF02901	++	++	n/a	n/a	n/a
PF03061	-	-	0	-	0
PF03171	-	-	no MW	-	0
PF03332	-	-	n/a	n/a	n/a
PF03414	++C	+C	n/a	n/a	n/a

Success rate	**32/44**	**34/44**	**1/7**	**8/22**	**1/5**

ET: evolutionary trace method [13,14]; ConSurf: ConSurf method [9]; FASS, MB, S: three methods from the TreeDet package [32]. '++': SDPsite with both *λ *= 0.5 and *λ *= 1 performs significantly better (p-value < 0.01 in Mann-Whitney test), '+': SDPsite with either *λ *= 0.5 or *λ *= 1 performs significantly better, 'C': SDPsite with CPs only performs significantly better; 'n/a' method is not applicable, 'no MW': Mann-Whitney test is unreliable (less than 5 value in one of the sets), 0: method applicable, but no positions predicted, prediction considered not successful.

## Discussion

We have presented a new approach for automated identification of functional sites in protein structures, SDPsite. Its main advantage is that it considers not only conservation, but also specific differences among proteins from a protein family. A number of approaches aim to predict specificity determinants [25-41] and/or conserved functionally important sites [9-24], but they almost never combine structural and sequence information. The closest attempt to apply this kind of analysis to prediction of functional sites was the Evolutionary Trace (ET) method [11,12]. However, the ET approach takes into account all positions conserved within groups that arise when cutting the phylogenetic tree of the family at a certain distance from the root, thus often leading to too many predicted positions. SDPsite also extracts all possible groups of proteins corresponding to different identity cutoffs, but, unlike ET, it selects the least probable to arise by chance, i.e. the most probable functional grouping.

We tested SDPsite on two benchmark datasets of enzyme families, for which details of function and binding mode of ligands were known, and specificity of proteins was, in one case, differing within the family, and in the other case, same for all proteins of the family. The tests show that for almost all cases SDPsite succeeds to predict the functional site (i.e. the active cleft of the enzyme) with a good accuracy. The average values of the minimal and average distances from the best cluster to the ligand over all families (Table 2) demonstrate that the predicted best cluster is compactly situated in the active site of the enzymes in most families. As noted above, the prediction of specificity determinants is the main novel feature of our method, but ignoring them and using only conserved positions to build the best cluster can lead to a decrease of the sensitivity of the method (Additional file 1, Table 3). Using this strategy some residues crucial for the function are missed (e.g., substrate-binding residues in tRNA-ribosyltransferases, see Results). Thus clusters that include SDPs often define a more practically useful set of functional residues. Comparisons to other methods for functional site predictions show that SDPsite is marginally or substantially better than the previous approaches, supporting the notion that the inclusion of specificity determining residues generally augments predictions of functional sites.

We provide predictions of functional sites for a large number of proteins, whose structures were solved in a high-throughput manner and lack functional annotation. In some cases (e.g. YCII-related domain, PHP domain, see Results section) we provide evidence that a family of homologous proteins includes proteins of different specificity and suggest composition of specificity groups. When specificity of some members of these groups is known, one can transfer it, with caution, to uncharacterized proteins, assisting their annotation (see the example of PHP domain).

## Conclusion

We have introduced a novel approach to identification of functionally important sites in protein structures. It bridges the gap between two existing categories of methods dealing with the same problem: sequence-centric comparative techniques and the structure-centric techniques that incorporate some physico-chemical considerations. Sequence data are already overwhelming, and structures lacking functional annotation are likely to increase in number for many years to come. Thus practical tools like that described will be of great benefit for those interested in inferring function directly from structure.

## Methods

### SDPsite algorithm

The approach takes as input the multiple sequence alignment of a protein family, the corresponding phylogenetic tree and a 3D structure of one of the family members. The algorithm is composed of three basic steps: (1) prediction of specificity determining positions (SDPs) (at this step, the sequences are automatically divided into a number of specificity groups, i.e. groups of proteins with presumably the same specificity); (2) prediction of conserved positions (CPs); and (3) identification of the best spatial cluster.

#### Prediction of specificity determining positions

We previously developed an algorithm for prediction of specificity determining positions (SDPs) [25]. Given a set of protein sequences divided into a number of specificity groups, this method searches for the positions of the alignment, for which the distribution of amino acids is best correlated with the specificity groups. We suppose that proteins from the same specificity group have the same specificity, and specificity differs for proteins from different groups. Thus, the identified positions are likely to confer the specific differences in the function.

To assess the correlation, we use *mutual information *of each position *p *of the alignment:



where *f*_*p*_(*α*, *i*) is the frequency of amino acid *α *in position *p *in specificity group *i*, *f*_*p*_(*α*)is frequency of the amino acid *α *in position *p *in the whole alignment, *f*(*i*) is the fraction of group *i *in the whole alignment. To account for the biological nature of the data, we introduce a number of corrections, which we discuss in detail in [25]. Then we calculate the mean  and the variance  of the expected mutual information and the statistical significance (Z-scores) for each position:



Then, to identify the number of highest-scoring positions, which are most likely to be SDPs, for each *k *positions with highest Z-scores, we compute the probability of getting this many Z-scores greater or equal to the smallest of the *k *by chance, assuming the normal distribution of the Z-scores. k* positions, for which this probability is the lowest, are the predicted SDPs:



where



The probability of these k* SDPs, P* = *P*{there are at least *k *cases: *Z *≥ *Z*_(*k*)_}, is called the statistical significance of the set of k* SDPs.

#### Determining specificity groups for a multiple sequence alignment

**Figure 9:**
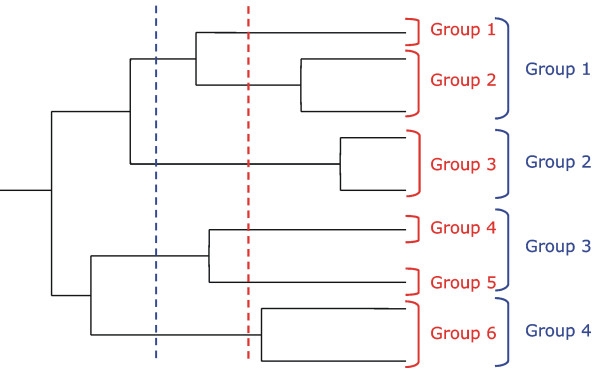
**Automated grouping procedure**. Two possible groupings are shown in red and in blue.

To allow large-scale application of SDPsite, we developed a procedure for automated identification of specificity groups from a multiple sequence alignment of the family alone. We applied the idea from Lichtarge and colleagues [11]: we place the root of the phylogenetic tree in the midpoint of the longest path between two leaves and consider the sets of groupings derived from cutting the tree on different distances from the root (Fig. 9). We ignore groups with fewer than three sequences. Then for each grouping we compute the SDPs as described above and select the grouping with the lowest P*, i.e. the least probable to arise by chance set of SDPs.

We need to correct for the fact that larger groups tend to produce larger Z-scores. When considering the same alignment and increasing the number of sequences in each group, Z-scores can be approximated by a log function (data not shown). Thus we divide Z-scores by the logarithm of the average number of sequences in a specificity group.

#### Prediction of conserved positions

For each position *p *of the alignment, we assessed its conservation using the Sander-Schneider conservation measure [57]:



where *N *is the number of sequences in the alignment, *d*(*s*_*i*_, *s*_*j*_) is the distance between sequences *s*_*i *_and *s*_*j*_, equal to , *s*_*i*_(*p*) and *s*_*j*_(*j*) is the amino acid in position *p *in sequence, *s*_*i *_and *s*_*j *_respectively, *M*(*α*, *β*) is the BLOSUM62 amino acid substitution matrix.

Analogous to SDPs, we calculate Z-score for each position:



 is the background distribution generated by 10000 times picking random positions from the alignment to form the random alignment column. This corresponds to conservation in a set of unaligned sequences. Since aligning itself introduces some bias, we correct for it by centering Z-scores: . Then we select the least probable set of CPs using procedures described in the previous section.

#### Construction of the best cluster

To compute SDPs and CPs we do not need the 3D structure of proteins of the family. We make use of it when constructing the best cluster for the first time. When there are resolved 3D structures for more than one protein of the family, the resulting best cluster may differ depending on the structure, but our tests show that it is usually not the case (data not shown).

We map the predicted SDPs and CPs onto the structure and construct the best cluster using the layered clustering based on tightness of set function algorithm [58] as follows. Consider a graph where vertices correspond to the predicted SDPs and CPs, and an edge connects every pair of vertices. Let *H*_0 _be the union of all vertices of the graph. Then for each *i *we calculate its weight: , where the sum is over all other vertices *j *from *H*_0_, *ω*_*ij *_is the weight of the edge connecting *i *and *j*, calculated as:



where *d*_*ij *_is the Euclidian distance between closest atoms of amino acids corresponding to vertices *i *and *j, R *= 5 Å is the average distance between centers of atoms in contact, *D *= 15 Å is the influence distance of the atom. *R *and *D *are empirical constants. *λ*_*i *_= 0.5 if the vertex corresponds to a CP, 1 otherwise. Thus CPs get twice as little weight as SDPs,. The rationale for this is the fact that in the benchmark datasets there is a clear tendency for more SDPs in the active site for families from the diverse dataset (10 SDPs on average closer than 10 Å to the ligand, opposed to 7.38 CPs). The equal measure derives from the fact that the homogeneous dataset has no such bias, (10.11 and 11.83 SDPs and CPs, respectively). For an unknown family then, it is logical to try both parameter settings.

We then find a subset *F*_0 _⊂ *H*_0_, for all vertices of which *μ *is minimal and equals . Let *H*_1 _= *H*_0_\*F*_0_. We repeat this until we get an empty set at some point. Thus we construct a series of layered clusters *H*_0 _∟ *H*_1 _∟ ... ∟ *H*_*N *_∟ Ψ. We select the cluster *H*_*n*_, for which  and call it *the best cluster*.

In certain cases we refer to a *second best cluster*. It is constructed analogous to the best cluster, after all amino acids from the best cluster are excluded from the initial set.

### Construction of the datasets

#### Benchmark datasets

To assess the performance of SDPsite, we built two benchmark datasets containing enzyme families with well-characterized function and at least one structure with bound natural ligands or their close analogs. The first dataset corresponds to the situation of a protein family, which acts on a variety of substrates, the second accounts for the case when all the proteins specificities are the same. We need these two datasets, because we do not know in advance to which of the two types the query family belongs.

We used alignments from the Pfam database [59]. The first dataset, termed *diverse*, consists of families, for which EC numbers differing in the last digit (usually accounting for the enzyme specificity) were assigned to at least two proteins. The second dataset was termed *homogeneous *and includes families, where all the proteins have the same EC number. The diverse dataset mimics the situation when the family in question contains proteins that bind different ligands (e.g. hydrolases of different specificity); the homogeneous dataset approximates sets of proteins that perform a single specific function in the cell (e.g. DNA polymerase), though (see Results) EC number is not necessarily sufficient to do this. The statistics and the bound ligands for these dataset are given in Tables 1 and 5.

To evaluate the performance of SDPsite for these datasets, we developed several statistical measures. Let *d*(*i*, *j*) be the distance between the closest atoms of amino acids *i *and *j *(*j *can represent the bound ligand or the set of bound ligands as well), *ψ *be a set of predicted residues, either all SDPs, all CPs, or the best cluster, *n*(Ψ) be the number of elements in Ψ. We calculate:

1) Minimal distance to the ligand: ;

2) Average distance to the ligand: ;

3) Diameter of the predicted set of SDPs, CPs and the best cluster: ;

4) Average pairwise distance between the residues within the predicted set: .

We also compute the significance of the average distance, i.e. the fraction of atoms that are located this close or closer to the ligand. This gives us an idea of which portion of the protein our prediction covers: the smaller the significance, the more precise the predictions.

#### Test dataset

We selected 193 Pfam families based on the following criteria: (1) the function of the family must be unknown or poorly characterized; (2) the family must include a protein with a 3D structure resolved in the framework of the Structural Genomics initiative or lack clear functional annotation; and (3) there should be no proteins with well-studied structure or function. A structure was considered as poorly annotated and coming from SG project, if the PDB file contained the words "structural genomics", "hypothetical protein" or "unknown function" in its HEADER or KEYWDS sections. Then we excluded families that consist of fewer than 6 sequences, because it not possible to predict SDPs for sets smaller than this size, and ended up with 124 families (listed in Additional file 2). All the alignments were downloaded from Pfam [59] and the corresponding phylogenetic trees from Pandit [60].

A web server to use SDPsite is available at:  and . The data for all predictions discussed is also available on this web site.

## Authors' contributions

OVK performed the collection of data, wrote the program, participated in the analysis and drafted the manuscript. MSG and RBR participated in the design of the study and the analysis of results, and helped to draft the manuscript. All authors read and approved the manuscript.

## Supplementary Material

Additional File 1 **Distance measures designed to assess the quality of the predictions of SDPsite**. A detailed list of different measures based on the distance between the predicted residues, designed to assess the quality of the prediction. Click here for file

Additional File 2 **Poorly characterized Pfam families, used in the analysis**. A list of all Pfam families included in the *de novo *prediction of functional site, grouped by function. Click here for file

Additional File 3 **Assessment of the quality of the predicions of SDPsite and other available methods**. A detailed comparison of performance of different methods for prediction of functional site, using same distance measures as in Additional file 1. Click here for file

## References

[B1] Holm L, Sander C (1996). Dali: a network tool for protein structure comparison. Trends Biochem Sci.

[B2] Taylor WR, Flores TP, Orengo CA (1994). Multiple protein structure alignment. Protein Sc.

[B3] Shindyalov IN, Bourne PE (1998). Protein structure alignment by incremental combinatorial extension (CE) of the optimal path. Protein Eng.

[B4] Kleywegt GJ (1999). Recognition of spatial motifs in protein structures. J Mol Biol.

[B5] Stark A, Russell RB (2003). Annotation in three dimensions. PINTS: Patterns in Non-homologous Tertiary Structures. Nucleic Acids Res.

[B6] Porter CT, Bartlett GJ, Thornton JM (2004). The Catalytic Site Atlas: a resource of catalytic sites and residues identified in enzymes using structural data. Nucleic Acids Res.

[B7] Dodson G, Wlodawer A (1998). Catalytic triads and their relatives. Trends Biochem Sci.

[B8] Stark A, Shkumatov A, Russell RB (2005). Finding functional sites in structural genomics proteins. Structure.

[B9] Landau M, Mayrose I, Rosenberg Y, Glaser F, Martz E, Pupko T, Ben-Tal N (2005). ConSurf 2005: the projection of evolutionary conservation scores of residues on protein structures. Nucleic Acids Res.

[B10] Aloy P, Querol E, Aviles FX, Sternberg MJ (2001). Automated structure-based prediction of functional sites in proteins: applications to assessing the validity of inheriting protein function from homology in genome annotation and to protein docking. J Mol Biol.

[B11] Lichtarge O, Bourne HR, Cohen FE (1996). An evolutionary trace method defined binding surfaces common to protein families. J Mol Biol.

[B12] Yao H, Kristensen DM, Mihalek I, Sowa ME, Shaw C, Kimmel M, Karvaki L, Lichtarge O (2003). An accurate, sensitive, and scalable method to identify functional sites in protein structures. J Mol Biol.

[B13] del Sol Mesa A, Pazos F, Valencia A (2003). Automatic methods for predicting functionally important residues. J Mol Biol.

[B14] Polacco BJ, Babbitt PC (2006). Automated discovery of 3D motifs for protein function annotation. Bioinformatics.

[B15] Capra J, Singh M (2007). Predicting functionally important residues from sequence conservation. Bioinformatics.

[B16] Manning JR, Jefferson ER, Barton GJ (2008). The contrasting properties of conservation and correlated phylogeny in protein functional residue prediction. BMC Bioinformatics.

[B17] Landgraf R, Xenarios I, Eisenberg D (2001). Three-dimensional cluster analysis identifies interfaces and functional residue clusters in proteins. J Mol Biol.

[B18] Ma B, Elkayam T, Wolfson H, Nussinov R (2003). Protein-protein interactions: structurally conserved residues distinguish between binding sites and exposed protein surfaces. Proc Natl Acad Sci USA.

[B19] Ahmad S, Keskin O, Sarai A, Nussinov R (2008). Protein-DNA interactions: structural, thermodynamic and clustering patterns of conserved residues in DNA-binding proteins. Nucleic Acids Res.

[B20] Shulman-Peleg A, Shatsky M, Nussinov R, Wolfson HJ (2008). Prediction of interacting single-stranded RNA bases by protein-binding patterns. J Mol Biol.

[B21] Hendlich M, Rippmann F, Barnickel G (1997). LIGSITE: automatic and efficient detection of potential small molecule-binding sites in proteins. J Mol Graph Model.

[B22] Laurie ATR, Jackson RM (2005). Q-SiteFinder: an energy-based method for the prediction of protein-ligand binding sites. Bioinformatics.

[B23] Koczyk G, Wyrwicz LS, Rychlewski L (2007). LigProf: a simple tool for in silico prediction of ligand-binding sites. J Mol Model.

[B24] Dundas J, Ouyang Z, Tseng J, Binowski A, Turpaz Y, Liang J (2006). CASTp: computed atlas of surface topography of proteins with atructural and topographical mapping of functionally annotated residues. Nucl Acids Res.

[B25] Kalinina OV, Mironov AA, Gelfand MS, Rakhmaninova AB (2004). Automated selection of positions determining functional specificity of proteins by comparative analysis of orthologous groups in protein families. Protein Sci.

[B26] Hannenhalli SS, Russell RB (2000). Analysis and prediction of functional sub-types from protein sequence alignments. J Mol Biol.

[B27] Mirny LA, Gelfand MS (2002). Using orthologous and paralogous proteins to identify specificity-determining residues in bacterial transcription factors. J Mol Biol.

[B28] Gaucher EA, Gu X, Miyamoto MM, Benner SA (2002). Predicting functional divergence in protein evolution by site-specific rate shifts. Trends Biochem Sci.

[B29] Pei J, Cai W, Kinch LN, Grishin NV (2006). Prediction of functional specificity determinants from protein sequences using log-likelihood ratios. Bioinformatics.

[B30] Carro A, Tress M, de Juan D, Pazos F, Lopez-Romero P, del Sol A, Valencia A, Rojas AM (2006). TreeDet: a web server to explore sequence space. Nucl Acids Res.

[B31] Donald JE, Shakhnovich EI (2005). Predicting specificity-determining residues in two large eukaryotic transcription factor families. Nucl Acids Res.

[B32] Chakrabarti S, Bryant SH, Panchenko AR (2007). Functional specificity lies within the properties and evolutionary changes of amino acids. J Mol Biol.

[B33] Ye K, Feenstra KA, Heringa J, IJzerman AP, Marchiori E (2008). Multi-RELIEF: a method to recognize specificity determining residues from multiple sequence alignments using a machine-learning approach for feature weighting. Bioinformatics.

[B34] Feenstra KA, Pirovano W, Krab K, Heringa J (2007). Sequence harmony: detecting functional specificity from alignments. Nucl Acids Res.

[B35] Reva B, Antipin Y, Sander C (2007). Determinants of protein function revealed by combinatorial entropy optimization. Genome Biol.

[B36] Wallace IM, Higgins DG (2007). Supervised multivariate analysis of sequence groups to identify specificity determining residues. BMC Bioinformatics.

[B37] Ye K, Vriend G, IJzerman AP (2008). Tracing evolutionary pressure. Bioinformatics.

[B38] Edwards RJ, Shields DC (2005). BADASP: predicting functional specificity in protein families using ancestral sequences. Bioinformatics.

[B39] Mayer KM, McCorkle SR, Shanklin J (2005). Linking enzyme sequence to function using conserved property difference locator to identify and annotate positions likely to control specific functionality. BMC Bioinformatics.

[B40] Capra JA, Singh M (2008). Characterization and prediction of residues determining protein functional specificity. Bioinformatics.

[B41] Sankararaman S, Sjolander K (2008). INTREPID – INformation-theoretic TREe traversal for Protein functional site IDentification. Bioinformatics.

[B42] Kalinina OV, Russell RB, Rakhmaninova AB, Gelfand MS (2007). Computational method for prediction of protein functional sites using specificity determinants. Mol Biol (Mosk).

[B43] Chandonia J-M, Brenner SE (2006). The impact of structural genomics: expectations and outcomes. Science.

[B44] Wu G, Fiser A, ter Kuile B, Sali A, Müller M (1999). Convergent evolution of Trichomonas vaginalis lactate dehydrogenase from malate dehydrogenase. Proc Natl Acad Sci USA.

[B45] Todd AE, Orengo CA, Thornton JM (2002). Plasticity of enzyme active sites. Trends Biochem Sci.

[B46] Romier C, Reuter K, Suck D, Ficner R (1996). Crystal structure of tRNA-guanine transglycosylase: RNA modification by base exchange. EMBO J.

[B47] Yeats C, Bentley S, Bateman A (2003). New knowledge from old: in silico discovery of novel protein domains in Streptomyces coelicolor. BMC Microbiol.

[B48] Willis MA, Song F, Zhuang Z, Krajewski W, Chalamasetty VR, Reddy P, Howard A, Dunaway-Mariano D, Herzberg O (2005). Structure of YciI from Haemophilus influenzae (HI0828) reveals a ferredoxin-like alpha/beta-fold with a histidine/aspartate centered catalytic site. Proteins.

[B49] Rodionov DA, Vitreschak AG, Mironov AA, Gelfand MS (2003). Comparative genomics of the vitamin B12 metabolism and regulation in prokaryotes. J Biol Chem.

[B50] Maier T, Jacobi A, Sauter M, Böck A (1993). The product of the hypB gene, which is required for nickel incorporation into hydrogenases, is a novel guanine nucleotide-binding protein. J Bacteriol.

[B51] Zambelli B, Musiani F, Savini M, Tucker P, Ciurli S (2007). Biochemical studies on Mycobacterium tuberculosis UreG and comparative modeling reveal structural and functional conservation among the bacterial UreG family. Biochemistry.

[B52] Khil PP, Obmolova G, Teplyakov A, Howard AJ, Gilliland GL, Camerini-Otero RD (2004). Crystal structure of the Escherichia coli YjiA protein suggests a GTP-dependent regulatory function. Proteins.

[B53] Aravind L, Koonin EV (1998). Phosphoesterase domains associated with DNA polymerases of diverse origins. Nucleic Acids Res.

[B54] Teplyakov A, Obmolova G, Khil PP, Howard AJ, Camerini-Otero RD, Gilliland GL (2003). Crystal structure of the Escherichia coli YcdX protein reveals a trinuclear zinc active site. Proteins.

[B55] Khil PP, Camerini-Otero RD (2002). Over 1000 genes are involved in the DNA damage response of Escherichia coli. Mol Microbiol.

[B56] Pupko T, Bell RE, Mayrose I, Glaser F, Ben-Tal N (2002). Rate4Site: an algorithmic tool for the identification of functional regions in proteins by surface mapping of evolutionary determinants within their homologues. Bioinformatics.

[B57] Valdar WSJ (2002). Scoring residue conservation. Proteins.

[B58] Mirkin B, Muchnik I (2002). Layered clusters of tightness set functions. Appl Math Lett.

[B59] Finn RD, Mistry J, Schuster-Böckler B, Griffiths-Jones S, Hollich V, Lassmann T, Moxon S, Marshall M, Khanna A, Durbin R, Eddy SR, Sonnhammer EL, Bateman A (2006). Pfam: clans, web tools and services. Nucleic Acids Res.

[B60] Whelan S, de Bakker PI, Quevillon E, Rodriguez N, Goldman N (2006). PANDIT: an evolution-centric database of protein and associated nucleotide domains with inferred trees. Nucl Acids Res.

