# Supplementary material for: Combining specificity determining and conserved residues improves functional site prediction
Source: BMC Bioinformatics. 2009 Jun 9;10:174. doi: 10.1186/1471-2105-10-174 (PMC2709924; doi:10.1186/1471-2105-10-174)
Supplement: Additional File 1 — Distance measures designed to assess the quality of the predictions of SDPsite. A detailed list of different measures based on the distance between the predicted residues, designed to assess the quality of the prediction. [file 1471-2105-10-174-S1.doc]

**Supplementary Table 1.** Distance measures designed to assess the quality of the predictions of SDPsite.

**A. λ = 0.5**

| **Diverse dataset** | | | | |
| --- | --- | --- | --- | --- |
|  | Minimal distance to the bound ligand | Average distance f to the bound ligand | Diameter (distance between to most distant atoms from the set) | Average distance between the residues of the set |
| PF00108 |  |  |  |  |
| SDPs | 3.180304 | 17.59437 | 69.74209 | 21.59587 |
| CPs | 3.34775 | 13.28459 | 53.74695 | 19.31921 |
| Best cluster | 5.635135 | 15.99488 | 31.56041 | 10.64422 |
| PF00128 |  |  |  |  |
| SDPs | 6.40706 | 12.75191 | 32.29604 | 13.07732 |
| CPs | 2.649871 | 7.536309 | 39.4916 | 11.7445 |
| Best cluster | 2.649871 | 8.837783 | 34.252 | 11.58603 |
| PF00132 |  |  |  |  |
| SDPs | 3.104054 | 6.805069 | 10.76518 | 2.30041 |
| CPs | 6.827974 | 11.46666 | 13.05659 | 4.740566 |
| Best cluster | 3.104054 | 6.80965 | 10.90806 | 2.986738 |
| PF00135 |  |  |  |  |
| SDPs | 3.515434 | 16.65118 | 46.79948 | 19.74718 |
| CPs | 3.908795 | 18.11275 | 47.93955 | 15.99327 |
| Best cluster | 15.8951 | 17.92996 | 13.17818 | 2.339233 |
| PF00215 |  |  |  |  |
| SDPs | 2.796554 | 10.18505 | 40.2886 | 15.70155 |
| CPs | 2.573059 | 6.097588 | 24.83746 | 9.087499 |
| Best cluster | 2.796554 | 8.738578 | 30.49522 | 10.11171 |
| PF00278 |  |  |  |  |
| SDPs | 3.165753 | 10.68629 | 26.21686 | 10.43624 |
| CPs | 3.320466 | 16.1757 | 33.034 | 13.67369 |
| Best cluster | 3.165753 | 5.724032 | 17.5412 | 5.602686 |
| PF00293 |  |  |  |  |
| SDPs | 3.023916 | 4.826768 | 18.86981 | 5.897019 |
| CPs | 3.358439 | 4.771435 | 13.56691 | 3.802854 |
| Best cluster | 3.023916 | 4.801617 | 20.37412 | 6.327833 |
| PF00348 |  |  |  |  |
| SDPs | 2.83794 | 14.73868 | 61.65073 | 21.403 |
| CPs | 2.422726 | 5.143243 | 26.01262 | 9.227958 |
| Best cluster | 2.422726 | 6.114915 | 31.57922 | 10.11567 |
| PF00351 |  |  |  |  |
| SDPs | 2.781888 | 11.4363 | 49.36145 | 20.52586 |
| CPs | 3.240738 | 10.74166 | 50.37266 | 18.56265 |
| Best cluster | 6.547406 | 8.879932 | 18.69452 | 6.203661 |
| PF00579 |  |  |  |  |
| SDPs | 3.398748 | 11.3688 | 48.22918 | 17.65004 |
| CPs | 2.737281 | 5.768914 | 22.99648 | 9.017829 |
| Best cluster | 2.737281 | 5.936299 | 24.77126 | 9.224788 |
| PF00583 |  |  |  |  |
| SDPs | 3.634651 | 3.634651 | 6.066407 | n/a |
| CPs | 3.002778 | 7.025704 | 18.70606 | 7.603908 |
| Best cluster | 8.665725 | 9.637005 | 7.397515 | 1.326346 |
| PF00590 |  |  |  |  |
| SDPs | 5.833287 | 9.804109 | 21.17746 | 8.097036 |
| CPs | 3.519971 | 7.601498 | 29.13898 | 9.608749 |
| Best cluster | 3.519971 | 6.718228 | 14.49231 | 4.945374 |
| PF00755 |  |  |  |  |
| SDPs | 2.936713 | 17.47746 | 74.98663 | 26.54445 |
| CPs | 2.768412 | 12.65474 | 51.42133 | 20.543 |
| Best cluster | 2.768412 | 9.726069 | 33.50854 | 9.734921 |
| PF00871 |  |  |  |  |
| SDPs | 5.378819 | 17.35659 | 65.37574 | 25.81136 |
| CPs | 2.976105 | 9.95393 | 33.57141 | 13.26213 |
| Best cluster | 2.976105 | 9.347339 | 20.34656 | 7.53557 |
| PF00896 |  |  |  |  |
| SDPs | 2.62215 | 13.73041 | 42.14978 | 15.50749 |
| CPs | 2.909455 | 8.316377 | 43.18687 | 15.46412 |
| Best cluster | 5.476151 | 12.70418 | 31.88756 | 11.76982 |
| PF00962 |  |  |  |  |
| SDPs | 3.747681 | 15.01044 | 55.436 | 19.95567 |
| CPs | 2.638306 | 7.059457 | 23.46695 | 7.459142 |
| Best cluster | 2.638306 | 11.55879 | 44.75675 | 15.35056 |
| PF01048 |  |  |  |  |
| SDPs | 3.400153 | 11.14172 | 43.76497 | 14.55778 |
| CPs | 3.056994 | 6.923563 | 27.79261 | 10.74666 |
| Best cluster | 3.400153 | 9.717405 | 29.33459 | 8.644557 |
| PF01112 |  |  |  |  |
| SDPs | 4.932907 | 14.33656 | 50.74458 | 18.84424 |
| CPs | 2.714922 | 9.327382 | 37.80039 | 14.67276 |
| Best cluster | 4.932907 | 11.02691 | 24.01174 | 6.820306 |
| PF01135 |  |  |  |  |
| SDPs | 2.745068 | 6.531537 | 32.07408 | 11.46062 |
| CPs | 2.748131 | 9.600185 | 28.29386 | 12.50613 |
| Best cluster | 2.748131 | 4.817747 | 24.65093 | 7.674836 |
| PF01202 |  |  |  |  |
| SDPs | 3.236404 | 10.62216 | 30.43881 | 12.66643 |
| CPs | 2.568921 | 3.362072 | 18.06049 | 6.341354 |
| Best cluster | 10.40789 | 13.26448 | 14.06922 | 2.997522 |
| PF01234 |  |  |  |  |
| SDPs | 3.169259 | 12.03649 | 35.77077 | 13.88407 |
| CPs | 2.506685 | 7.891168 | 40.2894 | 13.67481 |
| Best cluster | 3.169259 | 10.31858 | 28.27245 | 9.492839 |
| PF01467 |  |  |  |  |
| SDPs | 3.609188 | 9.117114 | 41.03492 | 13.56495 |
| CPs | 3.068755 | 4.278982 | 17.83779 | 6.533772 |
| Best cluster | 3.068755 | 7.463259 | 33.38001 | 10.42215 |
| PF01712 |  |  |  |  |
| SDPs | 2.386044 | 10.59032 | 43.77608 | 16.99166 |
| CPs | 2.643649 | 4.241307 | 34.05088 | 10.43515 |
| Best cluster | 2.386044 | 6.370525 | 28.42394 | 7.679302 |
| PF02274 |  |  |  |  |
| SDPs | 2.933131 | 9.55377 | 49.34938 | 15.74638 |
| CPs | 2.496145 | 4.718743 | 21.54685 | 7.777797 |
| Best cluster | 2.496145 | 5.85546 | 31.49234 | 9.5104 |
| PF03061 |  |  |  |  |
| SDPs | 2.82908 | 9.409062 | 26.60111 | 9.223427 |
| CPs | 3.130479 | 9.223483 | 31.79744 | 12.12124 |
| Best cluster | 2.82908 | 9.602468 | 23.73716 | 8.202892 |
| PF03171 |  |  |  |  |
| SDPs | 3.909563 | 7.408825 | 26.84617 | 10.13385 |
| CPs | 2.712503 | 3.595912 | 23.06681 | 6.650166 |
| Best cluster | 2.712503 | 6.294374 | 26.97735 | 9.807566 |
| PF03414 |  |  |  |  |
| SDPs | 2.84655 | 15.64079 | 61.69351 | 22.88781 |
| CPs | 2.576223 | 11.45475 | 44.32262 | 16.136 |
| Best cluster | 2.73832 | 10.63231 | 48.14249 | 16.26041 |
| **Homogeneous dataset** | | | | |
| PF00303 |  |  |  |  |
| SDPs | 3.181093 | 12.96701 | 50.53435 | 20.07229 |
| CPs | 1.900704 | 8.033908 | 44.74373 | 14.72316 |
| Best cluster | 13.4391 | 18.79655 | 15.59441 | 3.615434 |
| PF00693 |  |  |  |  |
| SDPs | 2.66796 | 15.45187 | 55.3564 | 20.7024 |
| CPs | 2.44871 | 10.07925 | 52.66126 | 16.93958 |
| Best cluster | 2.44871 | 13.96052 | 39.0569 | 13.95381 |
| PF00925 |  |  |  |  |
| SDPs | 3.654983 | 12.86593 | 53.41723 | 18.2992 |
| CPs | 2.594365 | 4.967259 | 36.557 | 10.53051 |
| Best cluster | 2.594365 | 12.13714 | 38.78272 | 13.29578 |
| PF01014 |  |  |  |  |
| SDPs | 3.232754 | 12.41133 | 51.14742 | 16.20534 |
| CPs | 3.648123 | 10.48268 | 37.0445 | 10.69375 |
| Best cluster | 3.232754 | 6.61737 | 22.73948 | 7.674514 |
| PF01227 |  |  |  |  |
| SDPs | 2.559442 | 6.692599 | 44.63691 | 12.98124 |
| CPs | 3.498652 | 5.859476 | 46.43928 | 22.65539 |
| Best cluster | 2.559442 | 6.651795 | 36.1263 | 11.40902 |
| PF01293 |  |  |  |  |
| SDPs | 3.479918 | 17.12673 | 67.5457 | 24.56025 |
| CPs | 2.551833 | 10.22843 | 54.49367 | 17.67056 |
| Best cluster | 2.940342 | 12.52186 | 51.74098 | 16.88742 |
| PF01583 |  |  |  |  |
| SDPs | 2.901673 | 10.49318 | 42.14594 | 15.93379 |
| CPs | 2.93993 | 6.425621 | 26.55115 | 10.46601 |
| Best cluster | 2.959602 | 11.98706 | 31.03261 | 10.52458 |
| PF01656 |  |  |  |  |
| SDPs | 3.374414 | 8.109039 | 18.3714 | 7.043305 |
| CPs | 2.575247 | 5.109007 | 22.76365 | 7.409558 |
| Best cluster | 2.575247 | 5.125436 | 21.00577 | 6.127506 |
| PF01702 |  |  |  |  |
| SDPs | 2.622804 | 7.668414 | 52.48916 | 19.55376 |
| CPs | 2.618472 | 10.47091 | 43.79381 | 17.48611 |
| Best cluster | 2.618472 | 5.524774 | 30.34204 | 10.93369 |
| PF01747 |  |  |  |  |
| SDPs | 2.668571 | 12.25881 | 58.01965 | 20.02898 |
| CPs | 2.701483 | 8.255392 | 53.9232 | 15.83519 |
| Best cluster | 2.668571 | 6.815191 | 40.81114 | 13.05693 |
| PF02110 |  |  |  |  |
| SDPs | 2.927812 | 11.23702 | 42.20861 | 16.57591 |
| CPs | 2.384361 | 7.408084 | 31.26064 | 12.6037 |
| Best cluster | 2.443342 | 8.321415 | 28.66518 | 8.692148 |
| PF02223 |  |  |  |  |
| SDPs | 2.978527 | 6.969228 | 43.15203 | 12.54032 |
| CPs | 2.748431 | 5.04949 | 29.97385 | 8.090575 |
| Best cluster | 3.363412 | 4.718588 | 14.13055 | 3.041869 |
| PF02277 |  |  |  |  |
| SDPs | 11.78923 | 13.16131 | 16.66676 | 11.06759 |
| CPs | 2.540307 | 6.982394 | 30.36991 | 10.46624 |
| Best cluster | 2.540307 | 8.827046 | 24.64266 | 9.914367 |
| PF02353 |  |  |  |  |
| SDPs | 3.105897 | 13.20014 | 51.57246 | 20.04875 |
| CPs | 2.758709 | 7.599444 | 41.87771 | 12.80202 |
| Best cluster | 2.758709 | 9.250346 | 46.55165 | 15.32893 |
| PF02569 |  |  |  |  |
| SDPs | 4.221899 | 14.26484 | 58.67531 | 21.28296 |
| CPs | 2.661906 | 8.193654 | 53.5792 | 14.77636 |
| Best cluster | 2.661906 | 8.673209 | 36.78794 | 13.82668 |
| PF02898 |  |  |  |  |
| SDPs | 6.286765 | 19.5817 | 70.61662 | 26.69705 |
| CPs | 2.835544 | 14.55749 | 68.72017 | 23.66958 |
| Best cluster | 6.685298 | 13.1211 | 31.87931 | 10.22721 |
| PF02901 |  |  |  |  |
| SDPs | 2.676026 | 18.33502 | 74.91845 | 24.64885 |
| CPs | 3.640352 | 20.69245 | 62.75972 | 25.90937 |
| Best cluster | 2.676026 | 8.799454 | 30.8115 | 8.704402 |
| PF03332 |  |  |  |  |
| SDPs | 7.37645 | 15.59898 | 57.45363 | 23.48739 |
| CPs | 2.917912 | 11.85059 | 51.25239 | 16.77415 |
| Best cluster | 7.349151 | 15.04964 | 24.2643 | 6.93945 |

* only one SDP identified

**B. λ = 1**

| **Diverse dataset** | | | | |
| --- | --- | --- | --- | --- |
|  | Minimal distance to the bound ligand | Average distance f to the bound ligand | Diameter (distance between to most distant atoms from the set) | Average distance between the residues of the set |
| PF00108 |  |  |  |  |
| SDPs | 3.835783 | 17.6934 | 65.72598 | 22.81345 |
| CPs | 3.180304 | 14.07826 | 56.59661 | 20.44622 |
| Best cluster | 7.705207 | 14.51476 | 20.59358 | 6.366878 |
| PF00128 |  |  |  |  |
| SDPs | 3.397821 | 11.77693 | 46.95904 | 15.36631 |
| CPs | 2.649871 | 7.536309 | 39.4916 | 11.7445 |
| Best cluster | 2.649871 | 7.396922 | 27.80934 | 8.697781 |
| PF00132 |  |  |  |  |
| SDPs | 15.76657 | 17.98227 | 10.76518 | 2.30041 |
| CPs | 5.505705 | 10.07509 | 13.05659 | 4.740566 |
| Best cluster | 12.41534 | 16.86889 | 10.90806 | 2.986738 |
| PF00135 |  |  |  |  |
| SDPs | 3.515434 | 18.29493 | 57.92635 | 23.94847 |
| CPs | 3.763561 | 16.72111 | 54.45672 | 20.0835 |
| Best cluster | 23.97454 | 26.45322 | 11.70679 | 2.457753 |
| PF00215 |  |  |  |  |
| SDPs | 2.796554 | 8.974107 | 38.00643 | 15.19433 |
| CPs | 2.573059 | 6.097588 | 24.83746 | 9.087499 |
| Best cluster | 2.86124 | 6.783322 | 19.42343 | 5.255305 |
| PF00278 |  |  |  |  |
| SDPs | 9.340968 | 22.07648 | 37.72748 | 13.07085 |
| CPs | 5.420693 | 22.0266 | 36.37283 | 14.92356 |
| Best cluster | 15.59058 | 24.85005 | 26.66093 | 9.57841 |
| PF00293 |  |  |  |  |
| SDPs | 3.502504 | 6.079274 | 17.08411 | 5.317226 |
| CPs | 3.358439 | 4.771435 | 13.56691 | 3.802854 |
| Best cluster | 3.358439 | 5.562157 | 20.33494 | 5.963018 |
| PF00348 |  |  |  |  |
| SDPs | 2.83794 | 14.66541 | 61.65073 | 21.12303 |
| CPs | 2.422726 | 5.143243 | 26.01262 | 9.227958 |
| Best cluster | 2.422726 | 6.114915 | 31.57922 | 10.11567 |
| PF00351 |  |  |  |  |
| SDPs | 2.781888 | 14.15262 | 49.86342 | 22.08423 |
| CPs | 3.240738 | 12.34424 | 50.37266 | 18.56265 |
| Best cluster | 2.781888 | 10.51953 | 36.98785 | 13.59281 |
| PF00579 |  |  |  |  |
| SDPs | 4.014546 | 11.82994 | 47.18998 | 18.10899 |
| CPs | 3.398748 | 7.510061 | 32.0525 | 11.54977 |
| Best cluster | 3.398748 | 6.928094 | 28.30262 | 10.23834 |
| PF00583 |  |  |  |  |
| SDPs | 3.634651 | 4.672125 | 12.2146 | 1.329797 |
| CPs | 3.395556 | 5.736093 | 21.90208 | 9.747084 |
| Best cluster | 3.634651 | 4.672125 | 12.2146 | 1.329797 |
| PF00590 |  |  |  |  |
| SDPs | 5.833287 | 9.804109 | 21.17746 | 8.097036 |
| CPs | 3.519971 | 7.601498 | 29.13898 | 9.608749 |
| Best cluster | 3.519971 | 6.405949 | 12.77071 | 4.325037 |
| PF00755 |  |  |  |  |
| SDPs | 3.444978 | 17.69604 | 74.99539 | 26.92491 |
| CPs | 2.71193 | 12.85871 | 53.19859 | 21.3357 |
| Best cluster | 2.768412 | 7.404852 | 34.45119 | 10.84897 |
| PF00871 |  |  |  |  |
| SDPs | 5.378819 | 17.61887 | 65.37574 | 25.75613 |
| CPs | 2.976105 | 9.95393 | 33.57141 | 13.26213 |
| Best cluster | 2.976105 | 9.324646 | 31.2211 | 10.8985 |
| PF00896 |  |  |  |  |
| SDPs | 2.62215 | 13.99971 | 42.14978 | 15.89273 |
| CPs | 2.909455 | 8.316377 | 43.18687 | 15.46412 |
| Best cluster | 2.62215 | 9.019356 | 36.58153 | 12.58866 |
| PF00962 |  |  |  |  |
| SDPs | 3.508429 | 15.22505 | 56.16686 | 20.83439 |
| CPs | 3.956923 | 8.472449 | 24.36439 | 8.253304 |
| Best cluster | 3.956923 | 8.812276 | 23.77265 | 8.449613 |
| PF01048 |  |  |  |  |
| SDPs | 3.400153 | 11.14172 | 43.76497 | 14.55778 |
| CPs | 3.056994 | 6.923563 | 27.79261 | 10.74666 |
| Best cluster | 3.056994 | 8.289002 | 26.63437 | 9.308493 |
| PF01112 |  |  |  |  |
| SDPs | 18.00103 | 29.77595 | 48.9748 | 18.66044 |
| CPs | 17.87254 | 24.30691 | 37.95356 | 14.63961 |
| Best cluster | 21.68901 | 28.32694 | 24.00691 | 6.550551 |
| PF01135 |  |  |  |  |
| SDPs | 2.745068 | 6.839192 | 36.7099 | 12.27809 |
| CPs | 2.748131 | 9.600185 | 28.29386 | 12.50613 |
| Best cluster | 2.748131 | 4.899454 | 24.65093 | 8.076042 |
| PF01202 |  |  |  |  |
| SDPs | 3.236404 | 9.75516 | 30.43881 | 12.45957 |
| CPs | 2.568921 | 3.362072 | 18.06049 | 6.341354 |
| Best cluster | 2.568921 | 3.717346 | 18.06049 | 6.674434 |
| PF01234 |  |  |  |  |
| SDPs | 3.169259 | 11.83695 | 35.77077 | 13.64406 |
| CPs | 2.506685 | 7.891168 | 40.2894 | 13.67481 |
| Best cluster | 2.687107 | 7.441918 | 24.33279 | 8.605025 |
| PF01467 |  |  |  |  |
| SDPs | 3.609188 | 9.954418 | 41.03492 | 14.30006 |
| CPs | 3.068755 | 4.278982 | 17.83779 | 6.533772 |
| Best cluster | 3.068755 | 7.696097 | 26.87908 | 8.349691 |
| PF01712 |  |  |  |  |
| SDPs | 2.386044 | 10.72454 | 43.77608 | 16.77124 |
| CPs | 2.643649 | 4.241307 | 34.05088 | 10.43515 |
| Best cluster | 2.386044 | 6.370525 | 28.42394 | 7.679302 |
| PF02274 |  |  |  |  |
| SDPs | 2.854305 | 11.01535 | 45.26833 | 16.66988 |
| CPs | 3.485472 | 6.483749 | 25.91887 | 11.05005 |
| Best cluster | 2.854305 | 6.0423 | 24.13431 | 8.939601 |
| PF03061 |  |  |  |  |
| SDPs | 2.82908 | 9.409062 | 26.60111 | 9.223427 |
| CPs | 3.130479 | 9.223483 | 31.79744 | 12.12124 |
| Best cluster | 2.82908 | 9.580103 | 23.73716 | 8.043682 |
| PF03171 |  |  |  |  |
| SDPs | 3.909563 | 7.408825 | 26.84617 | 10.13385 |
| CPs | 2.712503 | 3.595912 | 23.06681 | 6.650166 |
| Best cluster | 2.712503 | 6.294374 | 26.97735 | 9.807566 |
| PF03414 |  |  |  |  |
| SDPs | 2.84655 | 15.89106 | 61.69351 | 23.13621 |
| CPs | 2.576223 | 11.45475 | 44.32262 | 16.136 |
| Best cluster | 2.84655 | 8.786694 | 30.62238 | 10.03718 |
| **Homogeneous dataset** | | | | |
| PF00303 |  |  |  |  |
| SDPs | 3.181093 | 13.71673 | 50.53435 | 20.70248 |
| CPs | 1.900704 | 8.033908 | 44.74373 | 14.72316 |
| Best cluster | 3.787739 | 15.19242 | 22.89895 | 5.478964 |
| PF00693 |  |  |  |  |
| SDPs | 2.691726 | 13.03593 | 56.10982 | 20.86517 |
| CPs | 2.44871 | 10.07925 | 52.66126 | 16.93958 |
| Best cluster | 2.44871 | 4.679693 | 26.62663 | 9.40081 |
| PF00925 |  |  |  |  |
| SDPs | 3.654983 | 12.40226 | 53.41723 | 18.34123 |
| CPs | 2.594365 | 4.967259 | 36.557 | 10.53051 |
| Best cluster | 2.594365 | 11.55049 | 40.80558 | 13.59211 |
| PF01014 |  |  |  |  |
| SDPs | 3.232754 | 12.05093 | 51.14742 | 16.52198 |
| CPs | 3.648123 | 10.48268 | 37.0445 | 10.69375 |
| Best cluster | 3.648123 | 6.75474 | 17.16583 | 4.159905 |
| PF01227 |  |  |  |  |
| SDPs | 2.559442 | 6.463542 | 44.63691 | 14.35341 |
| CPs | 3.498652 | 5.859476 | 46.43928 | 22.65539 |
| Best cluster | 2.79603 | 4.942414 | 21.03244 | 6.294564 |
| PF01293 |  |  |  |  |
| SDPs | 3.479918 | 17.2591 | 67.5457 | 24.70971 |
| CPs | 2.551833 | 10.22843 | 54.49367 | 17.67056 |
| Best cluster | 2.551833 | 10.83364 | 44.10178 | 14.29064 |
| PF01583 |  |  |  |  |
| SDPs | 2.881637 | 10.52369 | 42.14594 | 16.33285 |
| CPs | 2.93993 | 6.425621 | 26.55115 | 10.46601 |
| Best cluster | 5.551062 | 13.94752 | 24.29435 | 9.120882 |
| PF01656 |  |  |  |  |
| SDPs | 2.575247 | 6.79013 | 18.3714 | 6.896637 |
| CPs | 2.575247 | 5.109007 | 22.76365 | 7.409558 |
| Best cluster | 2.575247 | 4.886196 | 21.36756 | 6.911315 |
| PF01702 |  |  |  |  |
| SDPs | 2.622804 | 7.430726 | 52.48916 | 19.09645 |
| CPs | 2.618472 | 10.47091 | 43.79381 | 17.48611 |
| Best cluster | 2.618472 | 6.261418 | 27.58975 | 10.40708 |
| PF01747 |  |  |  |  |
| SDPs | 2.668571 | 11.07907 | 58.01965 | 18.4796 |
| CPs | 2.701483 | 8.255392 | 53.9232 | 15.83519 |
| Best cluster | 2.668571 | 5.905208 | 40.81114 | 10.67783 |
| PF02110 |  |  |  |  |
| SDPs | 2.927812 | 10.60302 | 41.1222 | 16.09839 |
| CPs | 2.384361 | 7.408084 | 31.26064 | 12.6037 |
| Best cluster | 2.384361 | 7.184362 | 31.17385 | 10.31593 |
| PF02223 |  |  |  |  |
| SDPs | 2.978527 | 6.969228 | 43.15203 | 12.54032 |
| CPs | 2.748431 | 5.04949 | 29.97385 | 8.090575 |
| Best cluster | 2.748431 | 4.388735 | 25.00809 | 8.167529 |
| PF02277 |  |  |  |  |
| SDPs | 11.78923 | 13.16131 | 16.66676 | 11.06759 |
| CPs | 2.540307 | 6.982394 | 30.36991 | 10.46624 |
| Best cluster | 2.540307 | 8.827046 | 24.64266 | 9.914367 |
| PF02353 |  |  |  |  |
| SDPs | 3.105897 | 10.84722 | 50.69222 | 19.09277 |
| CPs | 2.758709 | 7.599444 | 41.87771 | 12.80202 |
| Best cluster | 2.758709 | 5.991554 | 25.78942 | 9.005108 |
| PF02569 |  |  |  |  |
| SDPs | 4.221899 | 13.76586 | 54.70875 | 20.93483 |
| CPs | 2.661906 | 8.193654 | 53.5792 | 14.77636 |
| Best cluster | 2.661906 | 8.673209 | 36.78794 | 13.82668 |
| PF02898 |  |  |  |  |
| SDPs | 6.183382 | 17.44288 | 70.77791 | 26.36047 |
| CPs | 2.835544 | 14.55749 | 68.72017 | 23.66958 |
| Best cluster | 3.10995 | 12.26547 | 34.04068 | 12.01154 |
| PF02901 |  |  |  |  |
| SDPs | 2.676026 | 18.12161 | 74.91845 | 24.42412 |
| CPs | 3.640352 | 20.69245 | 62.75972 | 25.90937 |
| Best cluster | 2.676026 | 6.81224 | 28.10267 | 8.469428 |
| PF03332 |  |  |  |  |
| SDPs | 8.263348 | 17.58875 | 64.17373 | 26.3017 |
| CPs | 2.917912 | 11.85059 | 51.25239 | 16.77415 |
| Best cluster | 2.917912 | 11.84671 | 55.60698 | 17.36516 |

C. No SDPs

| **Diverse dataset** | | | | |
| --- | --- | --- | --- | --- |
|  | Minimal distance to the bound ligand | Average distance f to the bound ligand | Diameter (distance between to most distant atoms from the set) | Average distance between the residues of the set |
| PF00108 |  |  |  |  |
| CPs | 3.34775 | 13.28459 | 53.74695 | 19.31921 |
| Best cluster | 2.774695 | 9.076996 | 36.79801 | 14.83092 |
| PF00128 |  |  |  |  |
| CPs | 2.649871 | 7.536309 | 39.4916 | 11.7445 |
| Best cluster | 2.649871 | 6.753954 | 23.07231 | 7.079584 |
| PF00132 |  |  |  |  |
| CPs | 5.505705 | 10.07509 | 13.05659 | 4.740566 |
| Best cluster | 5.505705 | 10.07509 | 13.05659 | 4.740566 |
| PF00135 |  |  |  |  |
| CPs | 3.908795 | 18.11275 | 47.93955 | 15.99327 |
| Best cluster | 8.443076 | 17.1894 | 24.38324 | 7.256834 |
| PF00215 |  |  |  |  |
| CPs | 2.573059 | 6.097588 | 24.83746 | 9.087499 |
| Best cluster | 2.86124 | 3.241366 | 8.96141 | 2.568871 |
| PF00278 |  |  |  |  |
| CPs | 3.320466 | 16.1757 | 33.034 | 13.67369 |
| Best cluster | 3.320466 | 6.027376 | 10.67503 | 2.342041 |
| PF00293 |  |  |  |  |
| CPs | 3.358439 | 4.771435 | 13.56691 | 3.802854 |
| Best cluster | 3.716202 | 5.124684 | 12.92095 | 2.708166 |
| PF00348 |  |  |  |  |
| CPs | 2.422726 | 5.143243 | 26.01262 | 9.227958 |
| Best cluster | 2.422726 | 4.909771 | 25.86659 | 7.867024 |
| PF00351 |  |  |  |  |
| CPs | 3.240738 | 12.34424 | 50.37266 | 18.56265 |
| Best cluster | 3.547119 | 10.93473 | 40.90345 | 14.23431 |
| PF00579 |  |  |  |  |
| CPs | 2.737281 | 5.768914 | 22.99648 | 9.017829 |
| Best cluster | 2.737281 | 5.768914 | 22.99648 | 9.017829 |
| PF00583 |  |  |  |  |
| CPs | 3.002778 | 7.025704 | 18.70606 | 7.603908 |
| Best cluster | 8.665725 | 9.637005 | 7.397515 | 1.326346 |
| PF00590 |  |  |  |  |
| CPs | 3.519971 | 7.601498 | 29.13898 | 9.608749 |
| Best cluster | 3.519971 | 7.285873 | 15.88184 | 4.93584 |
| PF00755 |  |  |  |  |
| CPs | 2.768412 | 12.65474 | 51.42133 | 20.543 |
| Best cluster | 2.768412 | 5.426719 | 20.50711 | 5.073496 |
| PF00871 |  |  |  |  |
| CPs | 2.976105 | 9.95393 | 33.57141 | 13.26213 |
| Best cluster | 3.519081 | 4.933108 | 12.36031 | 3.777122 |
| PF00896 |  |  |  |  |
| CPs | 2.909455 | 8.316377 | 43.18687 | 15.46412 |
| Best cluster | 5.991707 | 7.191895 | 21.63135 | 9.347374 |
| PF00962 |  |  |  |  |
| CPs | 2.638306 | 7.059457 | 23.46695 | 7.459142 |
| Best cluster | 2.638306 | 5.562593 | 21.04548 | 5.826694 |
| PF01048 |  |  |  |  |
| CPs | 3.056994 | 6.923563 | 27.79261 | 10.74666 |
| Best cluster | 3.056994 | 3.167563 | 10.0014 | 1.33054 |
| PF01112 |  |  |  |  |
| CPs | 2.714922 | 9.327382 | 37.80039 | 14.67276 |
| Best cluster | 10.52124 | 10.93287 | 6.769631 | 3.058663 |
| PF01135 |  |  |  |  |
| CPs | 2.748131 | 9.600185 | 28.29386 | 12.50613 |
| Best cluster | 12.77063 | 17.81891 | 16.09601 | 5.14152 |
| PF01202 |  |  |  |  |
| CPs | 2.568921 | 3.362072 | 18.06049 | 6.341354 |
| Best cluster | 2.784952 | 2.980107 | 7.038443 | 1.309263 |
| PF01234 |  |  |  |  |
| CPs | 2.506685 | 7.891168 | 40.2894 | 13.67481 |
| Best cluster | 2.506685 | 3.218843 | 16.8568 | 4.980302 |
| PF01467 |  |  |  |  |
| CPs | 3.068755 | 4.278982 | 17.83779 | 6.533772 |
| Best cluster | 3.621878 | 3.809908 | 6.819694 | 1.331115 |
| PF01712 |  |  |  |  |
| CPs | 2.643649 | 4.241307 | 34.05088 | 10.43515 |
| Best cluster | 2.643649 | 3.159105 | 27.30273 | 8.418592 |
| PF02274 |  |  |  |  |
| CPs | 2.496145 | 4.718743 | 21.54685 | 7.777797 |
| Best cluster | 2.496145 | 3.956804 | 21.54685 | 6.801163 |
| PF02668 |  |  |  |  |
| CPs | 2.928327 | 10.37945 | 44.07762 | 15.38884 |
| Best cluster | 2.928327 | 5.432023 | 19.50876 | 5.419728 |
| PF03061 |  |  |  |  |
| CPs | 3.130479 | 9.223483 | 31.79744 | 12.12124 |
| Best cluster | 10.67624 | 12.01401 | 8.418459 | 1.329519 |
| PF03171 |  |  |  |  |
| CPs | 2.712503 | 3.595912 | 23.06681 | 6.650166 |
| Best cluster | 3.405249 | 4.037617 | 9.735314 | 3.414464 |
| PF03414 |  |  |  |  |
| CPs | 2.576223 | 13.76657 | 44.32262 | 16.136 |
| Best cluster | 9.491412 | 14.67107 | 17.39919 | 4.104415 |
| **Homogeneous dataset** | | | | |
| PF00303 |  |  |  |  |
| CPs | 1.900704 | 8.033908 | 44.74373 | 14.72316 |
| Best cluster | 1.900704 | 6.008379 | 22.67732 | 7.330301 |
| PF00693 |  |  |  |  |
| CPs | 2.44871 | 10.07925 | 52.66126 | 16.93958 |
| Best cluster | 2.44871 | 4.869819 | 26.71499 | 8.587272 |
| PF00925 |  |  |  |  |
| CPs | 2.594365 | 4.967259 | 36.557 | 10.53051 |
| Best cluster | 2.594365 | 3.747018 | 29.22683 | 7.286052 |
| PF01014 |  |  |  |  |
| CPs | 3.648123 | 10.48268 | 37.0445 | 10.69375 |
| Best cluster | 3.91409 | 7.743419 | 14.5267 | 2.500458 |
| PF01227 |  |  |  |  |
| CPs | 3.498652 | 5.859476 | 46.43928 | 22.65539 |
| Best cluster | 5.551218 | 6.885042 | 8.927562 | 3.53802 |
| PF01293 |  |  |  |  |
| CPs | 2.551833 | 10.22843 | 54.49367 | 17.67056 |
| Best cluster | 2.940342 | 7.797042 | 30.17121 | 9.500263 |
| PF01583 |  |  |  |  |
| CPs | 2.93993 | 6.425621 | 26.55115 | 10.46601 |
| Best cluster | 2.93993 | 6.492122 | 26.24534 | 9.104468 |
| PF01656 |  |  |  |  |
| CPs | 2.575247 | 5.109007 | 22.76365 | 7.409558 |
| Best cluster | 2.575247 | 4.662829 | 21.36756 | 6.47332 |
| PF01702 |  |  |  |  |
| CPs | 2.618472 | 10.47091 | 43.79381 | 17.48611 |
| Best cluster | 12.44381 | 16.20996 | 10.60047 | 3.350914 |
| PF01747 |  |  |  |  |
| CPs | 2.701483 | 8.255392 | 53.9232 | 15.83519 |
| Best cluster | 2.701483 | 5.702344 | 28.12112 | 9.207788 |
| PF02110 |  |  |  |  |
| CPs | 2.384361 | 7.408084 | 31.26064 | 12.6037 |
| Best cluster | 2.443342 | 4.260174 | 18.63032 | 6.389579 |
| PF02223 |  |  |  |  |
| CPs | 2.748431 | 5.04949 | 29.97385 | 8.090575 |
| Best cluster | 2.748431 | 4.40288 | 12.80765 | 3.911525 |
| PF02277 |  |  |  |  |
| CPs | 2.540307 | 6.982394 | 30.36991 | 10.46624 |
| Best cluster | 3.650089 | 16.44641 | 57.17376 | 26.72304 |
| PF02353 |  |  |  |  |
| CPs | 2.758709 | 7.599444 | 41.87771 | 12.80202 |
| Best cluster | 2.773875 | 9.989449 | 39.85492 | 17.65876 |
| PF02569 |  |  |  |  |
| CPs | 2.661906 | 8.193654 | 53.5792 | 14.77636 |
| Best cluster | 2.661906 | 6.194906 | 30.33707 | 10.55669 |
| PF02898 |  |  |  |  |
| CPs | 2.835544 | 13.92722 | 65.66281 | 22.81602 |
| Best cluster | 6.183382 | 12.48581 | 26.52836 | 7.617948 |
| PF02901 |  |  |  |  |
| CPs | 3.640352 | 20.69245 | 62.75972 | 25.90937 |
| Best cluster | 20.44084 | 25.74178 | 42.17283 | 11.63848 |
| PF03332 |  |  |  |  |
| CPs | 2.917912 | 11.85059 | 51.25239 | 16.77415 |
| Best cluster | 7.349151 | 15.04964 | 24.2643 | 6.93945 |
