# Supplementary material for: Combining specificity determining and conserved residues improves functional site prediction
Source: BMC Bioinformatics. 2009 Jun 9;10:174. doi: 10.1186/1471-2105-10-174 (PMC2709924; doi:10.1186/1471-2105-10-174)
Supplement: Additional File 2 — Poorly characterized Pfam families, used in the analysis. A list of all Pfam families included in the de novo prediction of functional site, grouped by function. [file 1471-2105-10-174-S2.doc]

**Supplementary Table 2.** Poorly characterized Pfam families, used in the analysis.

| Function | Family ID | SDP+CP/length | Best cluster/length |
| --- | --- | --- | --- |
| Enzymes | PF01026 | 0.007947 | 0.002649 |
|  | PF01075 | 0.239583 | 0.072917 |
|  | PF01171 | 0.084291 | 0.042146 |
|  | PF01256 | 0.213058 | 0.054983 |
|  | PF01620 | 0.18408 | 0.054726 |
|  | PF01689 | 0.244813 | 0.06639 |
|  | PF01791 | 0.273381 | 0.036364 |
|  | PF01850 | 0.050691 | 0.02765 |
|  | PF01876 | 0.275 | 0.11875 |
|  | PF01903 | 0.073446 | 0.022599 |
|  | PF01923 | 0.175355 | 0.094787 |
|  | PF01928 | 0.026578 | 0.036545 |
|  | PF01965 | 0.036036 | 0.022523 |
|  | PF01981 | 0.227723 | 0.158416 |
|  | PF02126 | 0.053371 | 0.039326 |
|  | PF02367 | 0.210884 | 0.081633 |
|  | PF02381 | 0.25641 | 0.153846 |
|  | PF02492 | 0.131034 | 0.044828 |
|  | PF02502 | 0.309524 | 0.238095 |
|  | PF02527 | 0.321951 | 0.160976 |
|  | PF02580 | 0.333333 | 0.056911 |
|  | PF02585 | 0.026022 | 0.022305 |
|  | PF02627 | 0.038095 | 0.019048 |
|  | PF02811 | 0.015177 | 0.003373 |
|  | PF03009 | 0.132287 | 0.073991 |
|  | PF03130 | 0.170732 | 0.170732 |
|  | PF03320 | 0.175595 | 0.026786 |
|  | PF03364 | 0.033708 | 0.018727 |
|  | PF03641 | 0.071823 | 0.033149 |
|  | PF03652 | 0.307692 | 0.153846 |
|  | PF03737 | 0.235294 | 0.117647 |
|  | PF03747 | 0.177874 | 0.060738 |
|  | PF03795 | 0.065934 | 0.054945 |
|  | PF03972 | 0.234469 | 0.03006 |
|  | PF04095 | 0.145963 | 0.046584 |
|  | PF04227 | 0.069697 | 0.045455 |
|  | PF04371 | 0.229219 | 0.06801 |
|  | PF04452 | 0.155556 | 0.02963 |
|  | PF04471 | 0.165517 | 0.089655 |
|  | PF04509 | 0.15 | 0.075 |
|  | PF04564 | 0.084337 | 0.120482 |
|  | PF04673 | 0.287129 | 0.09901 |
|  | PF04960 | 0.142384 | 0.062914 |
|  | PF05138 | 0.171598 | 0.06213 |
|  | PF05161 | 0.233871 | 0.096774 |
|  | PF05175 | 0.084577 | 0.024876 |
|  | PF05544 | 0.142045 | 0.039773 |
|  | PF05899 | 0.214286 | 0.142857 |
|  | PF06185 | 0.239796 | 0.117347 |
|  | PF06821 | 0.21256 | 0.033816 |
|  | PF07683 | 0.051813 | 0.020725 |
|  | PF07876 | 0.037037 | 0.02963 |
|  | PF07883 | 0.03 | 0.02 |
|  | PF07972 | 0.234043 | 0.12766 |
| Transcription factors | PF01614 | 0.18932 | 0.092233 |
|  | PF02082 | 0.074534 | 0.018634 |
|  | PF02362 | 0.028249 | 0.039548 |
|  | PF02599 | 0.257576 | 0.151515 |
|  | PF03110 | 0.234568 | 0.08642 |
| Involved in translation | PF00736 | 0.284211 | 0.073684 |
|  | PF01300 | 0.095833 | 0.041667 |
|  | PF01912 | 0.2 | 0.038095 |
| Participate in cellular processes in a not completely understood fashion | PF00313 | 0.184211 | 0.118421 |
|  | PF00582 | 0.041667 | 0.010417 |
|  | PF01722 | 0.179775 | 0.044944 |
|  | PF01798 | 0.050633 | 0.012658 |
|  | PF01895 | 0.191919 | 0.141414 |
|  | PF01984 | 0.116071 | 0.0625 |
|  | PF01985 | 0.329897 | 0.247423 |
|  | PF02171 | 0.125307 | 0.029484 |
|  | PF02579 | 0.067901 | 0.049383 |
|  | PF03475 | 0.264151 | 0.188679 |
|  | PF03838 | 0.288235 | 0.141176 |
|  | PF04264 | 0.259091 | 0.127273 |
|  | PF05164 | 0.107914 | 0.05036 |
|  | PF06954 | 0.276786 | 0.116071 |
|  | PF07554 | 0.083333 | 0.027778 |
| Function unknown | PF01139 | 0.078809 | 0.022767 |
|  | PF01205 | 0.12069 | 0.043103 |
|  | PF01206 | 0.236842 | 0.118421 |
|  | PF01398 | 0.025381 | 0.010152 |
|  | PF01399 | 0.069959 | 0.032922 |
|  | PF01458 | 0.270916 | 0.087649 |
|  | PF01709 | 0.273381 | 0.115108 |
|  | PF01725 | 0.169811 | 0.037736 |
|  | PF01883 | 0.314607 | 0.202247 |
|  | PF01884 | 0.218254 | 0.09127 |
|  | PF01904 | 0.078035 | 0.043353 |
|  | PF01910 | 0.24 | 0.1 |
|  | PF01958 | 0.076271 | 0.025424 |
|  | PF01987 | 0.134545 | 0.083636 |
|  | PF02545 | 0.074561 | 0.026316 |
|  | PF02576 | 0.216667 | 0.05 |
|  | PF02590 | 0.150838 | 0.078212 |
|  | PF02594 | 0.216867 | 0.084337 |
|  | PF02598 | 0.157534 | 0.09589 |
|  | PF02635 | 0.223022 | 0.115108 |
|  | PF02637 | 0.100478 | 0.047847 |
|  | PF02657 | 0.3 | 0.046154 |
|  | PF03091 | 0.242718 | 0.048544 |
|  | PF03259 | 0.31068 | 0.135922 |
|  | PF03442 | 0.245098 | 0.127451 |
|  | PF03473 | 0.103604 | 0.040541 |
|  | PF03674 | 0.064516 | 0.024194 |
|  | PF03746 | 0.264591 | 0.070039 |
|  | PF03815 | 0.22314 | 0.090909 |
|  | PF03853 | 0.095 | 0.075 |
|  | PF03884 | 0.090909 | 0.075758 |
|  | PF03937 | 0.263736 | 0.164835 |
|  | PF03960 | 0.152 | 0.104 |
|  | PF04041 | 0.075472 | 0.040431 |
|  | PF04074 | 0.256098 | 0.182927 |
|  | PF04076 | 0.122302 | 0.064748 |
|  | PF04107 | 0.164134 | 0.088146 |
|  | PF04296 | 0.261364 | 0.090909 |
|  | PF04430 | 0.071429 | 0.047619 |
|  | PF04461 | 0.29878 | 0.036585 |
|  | PF04946 | 0.222222 | 0.118056 |
|  | PF05638 | 0.091463 | 0.036585 |
|  | PF05768 | 0.052632 | 0.032895 |
|  | PF06071 | 0.303371 | 0.213483 |
|  | PF06172 | 0.262295 | 0.163934 |
|  | PF06794 | 0.140845 | 0.042254 |
|  | PF07978 | 0.074074 | 0.055556 |
