# Supplementary material for: Combining specificity determining and conserved residues improves functional site prediction
Source: BMC Bioinformatics. 2009 Jun 9;10:174. doi: 10.1186/1471-2105-10-174 (PMC2709924; doi:10.1186/1471-2105-10-174)
Supplement: Additional File 3 — Assessment of the quality of the predicions of SDPsite and other available methods. A detailed comparison of performance of different methods for prediction of functional site, using same distance measures as in Additional file 1. [file 1471-2105-10-174-S3.doc]

**Supplementary Table 3.** Assessment of the quality of the predicions of SDPsite and other available methods. ET: evolutionary trace method [13, 14]; ConSurf: ConSurf method [9]; FASS, MB, S: three methods from the TreeDet package [32]. ‘n/a’: method not applicable to the alignment (the alignment does not meet the size/length conditions).

**A. Minimal distance to the ligand**

|  | SDPsite (λ = 0.5) | SDPsite (λ = 1) | SDPsite (no SDPs) | ET | rate4site | FASS | MB | S |
| --- | --- | --- | --- | --- | --- | --- | --- | --- |
| PF00108 | 5.635135 | 7.705207 | 2.774695 | 3.066793 | 3.34775 | 5.152707 | 3.272383 | 4.312686 |
| PF00128 | 2.649871 | 2.649871 | 2.649871 | 2.446471 | 4.003917 | n/a | n/a | n/a |
| PF00132 | 3.104054 | 12.41534 | 6.827974 | 3.104054 | 6.827974 | n/a | n/a | n/a |
| PF00135 | 15.8951 | 23.97454 | 8.443076 | 3.508849 | 5.896151 | n/a | n/a | n/a |
| PF00215 | 2.796554 | 2.86124 | 2.86124 | 2.573059 | 3.903579 | 2.796554 | 2.796554 | 2.83197 |
| PF00278 | 3.165753 | 15.59058 | 3.320466 | 3.165753 | 5.990025 | 3.180442 | 3.165753 | 3.320466 |
| PF00293 | 3.023916 | 3.358439 | 3.716202 | 2.982416 | 2.982416 | n/a | n/a | n/a |
| PF00303 | 13.4391 | 3.787739 | 1.900704 | 1.900704 | 3.150705 | 5.61841 | 3.150705 | n/a |
| PF00348 | 2.422726 | 2.422726 | 2.422726 | 2.422726 | 2.667125 | 2.83794 | 2.83794 | 2.83794 |
| PF00351 | 6.547406 | 2.781888 | 3.547119 | 2.663406 | 2.663406 | n/a | n/a | n/a |
| PF00579 | 2.737281 | 3.398748 | 2.737281 | 2.465308 | 2.465308 | 2.947659 | 3.398748 | 5.068665 |
| PF00583 | 8.665725 | 3.634651 | 8.665725 | 2.71022 | 3.468344 | n/a | n/a | n/a |
| PF00590 | 3.519971 | 3.519971 | 3.519971 | 2.604481 | 2.872523 | 5.755788 | 2.872523 | 2.872523 |
| PF00693 | 2.44871 | 2.44871 | 2.44871 | 2.44871 | 2.44871 | n/a | n/a | n/a |
| PF00755 | 2.768412 | 2.768412 | 2.768412 | 2.71193 | 3.655889 | n/a | n/a | n/a |
| PF00871 | 2.976105 | 2.976105 | 3.519081 | 2.725006 | 3.344687 | n/a | n/a | n/a |
| PF00896 | 5.476151 | 2.62215 | 5.991707 | 2.62215 | 2.909455 | n/a | n/a | n/a |
| PF00925 | 2.594365 | 2.594365 | 2.594365 | 2.594365 | 2.594365 | n/a | 2.679477 | n/a |
| PF00962 | 2.638306 | 3.956923 | 2.638306 | 2.638306 | 3.018085 | 3.589434 | 4.329836 | 3.33952 |
| PF01014 | 3.232754 | 3.648123 | 3.91409 | 3.006659 | 3.284498 | 39.80679 | 3.894174 | n/a |
| PF01048 | 3.400153 | 3.056994 | 3.056994 | 2.900974 | 3.400153 | 5.145068 | 3.400153 | 3.400153 |
| PF01112 | 4.932907 | 21.68901 | 10.52124 | 2.707508 | 2.707508 | n/a | n/a | n/a |
| PF01135 | 2.748131 | 2.748131 | 12.77063 | 2.616326 | 2.587536 | n/a | n/a | n/a |
| PF01202 | 10.40789 | 2.568921 | 2.784952 | 2.306628 | 2.638711 | 9.663414 | 2.336979 | n/a |
| PF01227 | 2.559442 | 2.79603 | 5.551218 | 2.559442 | 2.559442 | 2.559442 | 2.779815 | n/a |
| PF01234 | 3.169259 | 2.687107 | 2.506685 | 2.506685 | 2.506685 | n/a | n/a | n/a |
| PF01293 | 2.940342 | 2.551833 | 2.940342 | 2.322165 | 2.322165 | n/a | n/a | n/a |
| PF01467 | 3.068755 | 3.068755 | 3.621878 | 2.579968 | 3.068755 | n/a | 3.099449 | n/a |
| PF01583 | 2.959602 | 5.551062 | 2.93993 | 2.64085 | 2.879571 | 3.612451 | 2.64085 | n/a |
| PF01656 | 2.575247 | 2.575247 | 2.575247 | 2.575247 | 2.575247 | 2.575247 | 2.575247 | 2.575247 |
| PF01702 | 2.618472 | 2.618472 | 12.44381 | 2.491551 | 2.618472 | n/a | n/a | n/a |
| PF01712 | 2.386044 | 2.386044 | 2.643649 | 2.386044 | 2.386044 | n/a | n/a | n/a |
| PF01747 | 2.668571 | 2.668571 | 2.701483 | 2.668571 | 2.957757 | 2.668571 | 5.952965 | 4.120601 |
| PF02110 | 2.443342 | 2.384361 | 2.443342 | 2.384361 | 2.384361 | n/a | n/a | n/a |
| PF02223 | 3.363412 | 2.748431 | 2.748431 | 2.588637 | 2.660095 | 3.363412 | 2.660095 | n/a |
| PF02274 | 2.854305 | 2.854305 | 2.496145 | 2.496145 | 4.595961 | 2.922554 | 2.854305 | 2.922554 |
| PF02277 | 2.540307 | 2.540307 | 2.540307 | 2.525447 | 2.543802 | 11.98098 | 2.946876 | n/a |
| PF02353 | 2.758709 | 2.758709 | 2.773875 | 2.516214 | 2.773875 | n/a | n/a | n/a |
| PF02569 | 2.661906 | 2.661906 | 2.661906 | 2.661906 | 2.856549 | n/a | n/a | n/a |
| PF02898 | 6.685298 | 3.10995 | 6.183382 | 2.772708 | 2.772708 | n/a | n/a | n/a |
| PF02901 | 2.676026 | 2.676026 | 20.44084 | 2.932854 | 2.676026 | n/a | n/a | n/a |
| PF03061 | 2.82908 | 2.82908 | 10.67624 | 3.380278 | 3.515419 | n/a | 2.82908 | n/a |
| PF03171 | 2.712503 | 2.712503 | 3.405249 | 2.841252 | 2.712503 | 2.841252 | 2.841252 | n/a |
| PF03332 | 7.349151 | 2.917912 | 7.349151 | 2.44309 | 2.44309 | n/a | n/a | n/a |
| PF03414 | 2.73832 | 2.84655 | 3.797831 | 2.576223 | 2.67913 | n/a | n/a | n/a |

B. Average distance to the ligand

|  | SDPsite (λ = 0.5) | SDPsite (λ = 1) | SDPsite (no SDPs) | ET | rate4site | FASS | MB | S |
| --- | --- | --- | --- | --- | --- | --- | --- | --- |
| PF00108 | 15.99488 | 14.51476 | 9.076996 | 15.62428 | 17.31483 | 13.59183 | 17.79349 | 5.76085 |
| PF00128 | 8.837783 | 7.396922 | 6.753954 | 25.97795 | 26.946 | n/a | n/a | n/a |
| PF00132 | 6.80965 | 16.86889 | 11.46666 | 12.93509 | 12.39409 | n/a | n/a | n/a |
| PF00135 | 17.92996 | 26.45322 | 17.1894 | 18.3316 | 19.5988 | n/a | n/a | n/a |
| PF00215 | 8.738578 | 6.783322 | 3.241366 | 10.0169 | 13.39277 | 6.662106 | 8.310354 | 2.83197 |
| PF00278 | 5.724032 | 24.85005 | 6.027376 | 14.49445 | 20.73449 | 10.65197 | 11.71471 | 3.320466 |
| PF00293 | 4.801617 | 5.562157 | 5.124684 | 12.38195 | 7.931022 | n/a | n/a | n/a |
| PF00303 | 18.79655 | 15.19242 | 6.008379 | 12.30158 | 12.60742 | 5.61841 | 10.33427 | n/a |
| PF00348 | 6.114915 | 6.114915 | 4.909771 | 13.38407 | 11.82777 | 10.65092 | 12.56408 | 8.312394 |
| PF00351 | 8.879932 | 10.51953 | 10.93473 | 14.31931 | 13.1605 | n/a | n/a | n/a |
| PF00579 | 5.936299 | 6.928094 | 5.768914 | 15.21548 | 10.77431 | 10.47049 | 12.24079 | 12.14748 |
| PF00583 | 9.637005 | 4.672125 | 9.637005 | 10.45682 | 10.71007 | n/a | n/a | n/a |
| PF00590 | 6.718228 | 6.405949 | 7.285873 | 11.79584 | 12.11668 | 5.755788 | 11.02784 | 8.644703 |
| PF00693 | 13.96052 | 4.679693 | 4.869819 | 16.54788 | 13.30222 | n/a | n/a | n/a |
| PF00755 | 7.404852 | 7.404852 | 5.426719 | 19.07015 | 23.11774 | n/a | n/a | n/a |
| PF00871 | 9.347339 | 9.324646 | 4.933108 | 16.0982 | 19.11773 | n/a | n/a | n/a |
| PF00896 | 12.70418 | 9.019356 | 7.191895 | 12.76536 | 11.90288 | n/a | n/a | n/a |
| PF00925 | 12.13714 | 11.55049 | 3.747018 | 8.411637 | 9.627588 | n/a | 6.983401 | n/a |
| PF00962 | 11.55879 | 8.812276 | 5.562593 | 14.47821 | 12.94846 | 15.95318 | 15.97644 | 12.48811 |
| PF01014 | 6.61737 | 6.75474 | 7.743419 | 19.02327 | 18.63347 | 39.80679 | 21.50861 | n/a |
| PF01048 | 9.717405 | 8.289002 | 3.167563 | 12.60637 | 13.47617 | 9.521013 | 11.52065 | 6.846118 |
| PF01112 | 11.02691 | 28.32694 | 10.93287 | 14.47656 | 14.544 | n/a | n/a | n/a |
| PF01135 | 4.817747 | 4.899454 | 17.81891 | 11.24987 | 12.49373 | n/a | n/a | n/a |
| PF01202 | 13.26448 | 3.717346 | 2.980107 | 9.838118 | 9.892185 | 15.17907 | 7.99911 | n/a |
| PF01227 | 6.651795 | 4.942414 | 6.885042 | 15.76824 | 15.26331 | 13.70056 | 15.74168 | n/a |
| PF01234 | 10.31858 | 7.441918 | 3.218843 | 11.94981 | 12.21806 | n/a | n/a | n/a |
| PF01293 | 12.52186 | 10.83364 | 7.797042 | 16.23084 | 14.47121 | n/a | n/a | n/a |
| PF01467 | 7.463259 | 7.696097 | 3.809908 | 6.789225 | 6.578262 | n/a | 9.239681 | n/a |
| PF01583 | 11.98706 | 13.94752 | 6.492122 | 32.4192 | 33.45771 | 19.9168 | 28.70377 | n/a |
| PF01656 | 5.125436 | 4.886196 | 4.662829 | 12.65325 | 12.28362 | 10.52797 | 11.94643 | 10.43994 |
| PF01702 | 5.524774 | 6.261418 | 16.20996 | 33.27169 | 34.14121 | n/a | n/a | n/a |
| PF01712 | 6.370525 | 6.370525 | 3.159105 | 14.07423 | 12.18504 | n/a | n/a | n/a |
| PF01747 | 6.815191 | 5.905208 | 5.702344 | 15.79212 | 20.17001 | 13.11651 | 15.53011 | 11.26788 |
| PF02110 | 8.321415 | 7.184362 | 4.260174 | 13.20157 | 12.16162 | n/a | n/a | n/a |
| PF02223 | 4.718588 | 4.388735 | 4.40288 | 12.75303 | 12.28898 | 6.480429 | 10.86749 | n/a |
| PF02274 | 6.968345 | 6.0423 | 3.956804 | 12.0901 | 17.6844 | 7.753322 | 10.97778 | 2.922554 |
| PF02277 | 8.827046 | 8.827046 | 4.174552 | 16.66205 | 16.33181 | 20.45672 | 15.62027 | n/a |
| PF02353 | 9.250346 | 5.991554 | 9.989449 | 12.11965 | 12.88305 | n/a | n/a | n/a |
| PF02569 | 8.673209 | 8.673209 | 6.194906 | 13.25976 | 12.84756 | n/a | n/a | n/a |
| PF02898 | 13.1211 | 12.26547 | 12.48581 | 14.97252 | 14.40733 | n/a | n/a | n/a |
| PF02901 | 8.799454 | 6.81224 | 25.74178 | 20.28091 | 20.23314 | n/a | n/a | n/a |
| PF03061 | 9.602468 | 9.580103 | 12.01401 | 15.32648 | 12.47839 | n/a | 8.870518 | n/a |
| PF03171 | 6.294374 | 6.294374 | 4.037617 | 9.330088 | 7.797527 | 2.841252 | 8.230513 | n/a |
| PF03332 | 15.04964 | 11.84671 | 15.04964 | 14.35821 | 13.61553 | n/a | n/a | n/a |
| PF03414 | 10.63231 | 8.786694 | 8.84902 | 17.99426 | 17.0931 | n/a | n/a | n/a |

C. Significance of the average distance

|  | SDPsite (λ = 0.5) | SDPsite (λ = 1) | SDPsite (no SDPs) | ET | rate4site | FASS | MB | S |
| --- | --- | --- | --- | --- | --- | --- | --- | --- |
| PF00108 | 0.162566 | 0.122983 | 0.025075 | 0.152925 | 0.202545 | 0.10103 | 0.217443 | 0.004131 |
| PF00128 | 0.013412 | 0.006817 | 0.004826 | 0.01617 | 0.035217 | n/a | n/a | n/a |
| PF00132 | 0.007207 | 0.211082 | 0.055713 | 0.082926 | 0.072483 | n/a | n/a | n/a |
| PF00135 | 0.257762 | 0.654075 | 0.230516 | 0.272663 | 0.321538 | n/a | n/a | n/a |
| PF00215 | 0.062191 | 0.025597 | 4.47E-04 | 0.097026 | 0.231003 | 0.02375 | 0.052536 | 1.79E-04 |
| PF00278 | 0.005302 | 0.550221 | 0.006421 | 0.118527 | 0.344713 | 0.044801 | 0.060972 | 2.06E-04 |
| PF00293 | 0.003775 | 0.007146 | 0.005185 | 0.144477 | 0.027393 | n/a | n/a | n/a |
| PF00303 | 4.29E-01 | 0.250235 | 0.009941 | 0.137921 | 0.147788 | 0.007288 | 0.081272 | n/a |
| PF00348 | 0.005156 | 0.010208 | 0.00364 | 0.114018 | 0.072915 | 0.045486 | 0.090924 | 0.012129 |
| PF00351 | 0.053931 | 0.088502 | 0.099673 | 0.133425 | 0.095525 | n/a | n/a | n/a |
| PF00579 | 0.011898 | 0.02133 | 0.010407 | 0.265579 | 0.097949 | 0.089825 | 0.142375 | 0.139575 |
| PF00583 | 0.078571 | 0.004271 | 0.078954 | 0.056523 | 0.062683 | n/a | n/a | n/a |
| PF00590 | 0.022309 | 0.018984 | 0.029246 | 0.138433 | 0.14931 | 0.012499 | 0.114092 | 0.050878 |
| PF00693 | 0.178525 | 0.004147 | 0.004967 | 0.173027 | 0.092247 | n/a | n/a | n/a |
| PF00755 | 0.022493 | 0.008185 | 0.002431 | 0.181793 | 0.300688 | n/a | n/a | n/a |
| PF00871 | 0.030905 | 0.030673 | 0.002828 | 0.176237 | 0.301539 | n/a | n/a | n/a |
| PF00896 | 0.147515 | 0.05484 | 0.027264 | 0.149258 | 0.124251 | n/a | n/a | n/a |
| PF00925 | 0.224246 | 0.197094 | 0.008593 | 0.076472 | 0.116547 | n/a | 0.044756 | n/a |
| PF00962 | 0.092486 | 0.03722 | 0.006065 | 0.183529 | 0.131608 | 0.239781 | 0.240708 | 0.118003 |
| PF01014 | 0.012505 | 0.013453 | 0.021827 | 0.259085 | 0.252643 | 0.678881 | 0.305089 | n/a |
| PF01048 | 0.06769 | 0.040479 | 8.81E-05 | 0.151231 | 0.184607 | 0.063463 | 0.1163 | 0.021546 |
| PF01112 | 0.117799 | 0.355067 | 0.114639 | 0.248637 | 0.251782 | n/a | n/a | n/a |
| PF01135 | 0.006622 | 0.007438 | 0.431604 | 0.123979 | 0.168088 | n/a | n/a | n/a |
| PF01202 | 0.267432 | 0.001966 | 3.97E-04 | 0.036621 | 0.037876 | 0.301502 | 0.008784 | n/a |
| PF01227 | 0.020909 | 0.010837 | 0.022651 | 0.035373 | 0.017636 | 0.009701 | 0.012874 | n/a |
| PF01234 | 0.101112 | 0.03652 | 2.07E-04 | 0.158654 | 0.168684 | n/a | n/a | n/a |
| PF01293 | 0.086484 | 0.055097 | 0.017935 | 0.131086 | 0.095739 | n/a | n/a | n/a |
| PF01467 | 0.059284 | 0.065567 | 0.001854 | 0.042507 | 0.038203 | n/a | 0.122714 | n/a |
| PF01583 | 0.143537 | 0.220394 | 0.018014 | 0.017904 | 0.024232 | 1.68E-05 | 0.004929 | n/a |
| PF01656 | 0.007253 | 0.005642 | 0.004312 | 0.103616 | 0.091634 | 0.072131 | 0.092655 | 0.07291 |
| PF01702 | 6.99E-04 | 0.001177 | 0.040377 | 0.009663 | 0.007572 | n/a | n/a | n/a |
| PF01712 | 0.019209 | 0.019209 | 3.71E-04 | 0.095092 | 0.055825 | n/a | n/a | n/a |
| PF01747 | 0.010022 | 0.00571 | 0.004919 | 0.101075 | 0.222529 | 0.052708 | 0.091489 | 0.025863 |
| PF02110 | 0.03899 | 0.02344 | 0.002114 | 0.105577 | 0.082161 | n/a | n/a | n/a |
| PF02223 | 0.012386 | 0.010405 | 0.010441 | 0.089218 | 0.082947 | 0.005365 | 0.048736 | n/a |
| PF02274 | 0.011329 | 0.012567 | 0.002029 | 0.108547 | 0.30343 | 0.02906 | 0.083202 | 1.58E-04 |
| PF02277 | 0.045625 | 0.045625 | 0.00212 | 0.217142 | 0.201206 | 0.356614 | 0.163794 | n/a |
| PF02353 | 0.066083 | 0.014991 | 0.084915 | 0.151763 | 0.182086 | n/a | n/a | n/a |
| PF02569 | 0.050163 | 0.050163 | 0.01513 | 0.12607 | 0.103367 | n/a | n/a | n/a |
| PF02898 | 0.123365 | 0.099842 | 0.10554 | 0.182585 | 0.163296 | n/a | n/a | n/a |
| PF02901 | 0.024504 | 0.010198 | 0.535233 | 0.294104 | 0.291953 | n/a | n/a | n/a |
| PF03061 | 0.062084 | 0.061634 | 0.130285 | 0.244423 | 0.144888 | n/a | 0.047511 | n/a |
| PF03171 | 0.022818 | 0.022818 | 0.003534 | 0.099877 | 0.05378 | 7.68E-05 | 0.067071 | n/a |
| PF03332 | 0.231231 | 0.112519 | 0.231231 | 0.20869 | 0.179282 | n/a | n/a | n/a |
| PF03414 | 0.056546 | 0.028427 | 0.02915 | 0.160265 | 0.128505 | n/a | n/a | n/a |
